# Discovery of Novel Thiazole-Based SIRT2 Inhibitors as Anticancer Agents: Molecular Modeling, Chemical Synthesis and Biological Assays

**DOI:** 10.3390/ijms252011084

**Published:** 2024-10-15

**Authors:** Francesco Piacente, Giorgia Guccione, Naomi Scarano, Dario Lunaccio, Caterina Miro, Elena Abbotto, Annalisa Salis, Bruno Tasso, Monica Dentice, Santina Bruzzone, Elena Cichero, Enrico Millo

**Affiliations:** 1 Department of Experimental Medicine, Section of Biochemistry, University of Genoa, Viale Benedetto XV, 1, 16132 Genoa, Italy; francesco.piacente@unige.it (F.P.); giorgia.guccione@edu.unige.it (G.G.); dario.lunaccio@edu.unige.it (D.L.); elena.abbotto@unige.it (E.A.); annalisa.salis@unige.it (A.S.); enrico.millo@unige.it (E.M.); 2 Department of Pharmacy, Section of Medicinal Chemistry, School of Medical and Pharmaceutical Sciences, University of Genoa, Viale Benedetto XV, 3, 16132 Genoa, Italy; naomi.scarano@edu.unige.it (N.S.); bruno.tasso@unige.it (B.T.); 3 Department of Clinical Medicine & Surgery, University of Naples Federico II, Via S. Pansini, 5, 80131 Naples, Italy; caterinamiro87@gmail.com (C.M.); monica.dentice@unina.it (M.D.); 4 IRCCS Ospedale Policlinico San Martino, Largo Rosanna Benzi 10, 16132 Genoa, Italy

**Keywords:** SIRT2, sirtuin, drug design, molecular docking, enzymatic assays, inhibitor, biochemical assays, cancer

## Abstract

The search and development of effective sirtuin small molecule inhibitors (SIRTIs) continues to draw great attention due to their wide range of pharmacological applications. Based on SIRTs’ involvement in different biological pathways, their ligands were investigated for many diseases, such as cancer, neurodegenerative disorders, diabetes, cardiovascular diseases and autoimmune diseases. The elucidation of a substantial number of SIRT2–ligand complexes is steering the identification of novel and more selective modulators. Among them, SIRT2 in the presence of the SirReal2 analog series was the most studied. On this basis, we recently reported structure-based analyses leading to the discovery of thiazole-based compounds acting as SIRT2 inhibitors (**T1**, SIRT2 IC_50_ = 17.3 µM). Herein, ligand-based approaches followed by molecular docking simulations allowed us to evaluate in silico a novel small series of thiazoles (**3a**–**3d** and **5a**, **5d**) as putative SIRT2 inhibitors. Results from the computational studies revealed comparable molecular interaction fields (MIFs) and docking positionings of most of these compounds with respect to reference SIRT2Is. Biochemical and biological assays validated this study and pointed to compound **5a** (SIRT2 IC_50_ = 9.0 µM) as the most interesting SIRT2I that was worthy of further development as an anticancer agent.

## 1. Introduction

Sirtuins (SIRTs) are protein deacetylases classified as class III histone deacetylases (HDACs) [1]. This protein family catalyzes the removal of acetyl groups from ε-N-acetyl lysine amino acids of histone proteins, counteracting the activity of histone acetyltransferases (HATs) [2]. Dysregulation involving HDACs and HATs activities results in different disorders such as neurodegenerative syndromes, metabolic diseases, and cancer as related to different SIRT functions [3].

The class III HDAC family consists of seven members, named SIRT1-7, whose activity depends on NAD^+^ [4]. SIRTs have different subcellular localization and substrate specificity: SIRT1, SIRT6, and SIRT7 are predominantly nuclear proteins, SIRT2 is mostly in the cytoplasm and SIRT3–5 are located in mitochondria [5].

Because SIRTs are involved in different biological pathways, ranging from transcription to metabolism and genome stability, their dysregulation has been investigated in many diseases, such as cancer, neurodegenerative disorders, diabetes, and cardiovascular and autoimmune diseases [6,7]. Among them, SIRT1 and SIRT2, as well as their modulators, are the most studied [8,9].

To mention some of SIRT1 and SIRT2’s activities, SIRT1 plays an essential role in the maintenance of mitochondrial integrity by modulating the MEF2 transcription factors in the heart [10] and exerts longevity effects against aging-associated pathologies, metabolic dysfunction [11] and cardiovascular diseases [12]. SIRT2 is involved in several processes, including metabolism and also cell cycle regulation [13,14]. Upon a high-fat condition, the deletion of the *Sirt2* gene reduces muscle insulin sensitivity and contributes to liver insulin resistance in mice, potentially by affecting mitochondrial acetylation state [15].

Because SIRT2 is mainly expressed in the central nervous system (CNS), being overexpressed in neurological disease, it is thought to promote neurodegenerative events [16]. Accordingly, SIRT2 inhibition has been scouted to protect neurons from toxicity due to α-synuclein increased levels in Parkinson’s disease (PD) models [17]. In addition, SIRT2 expression is down- or up-regulated in different malignancies, making SIRT2 a druggable target for the development of anticancer agents [18,19].

**Figure 1:**
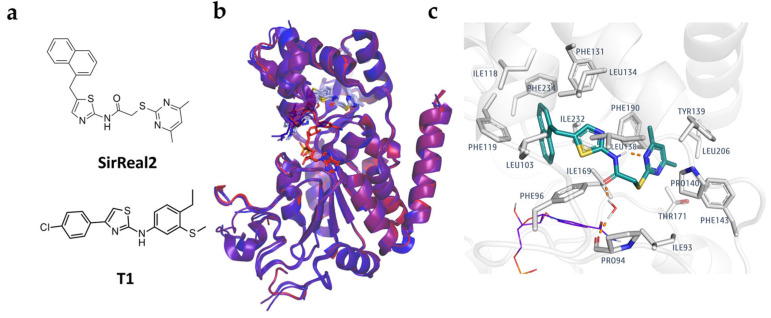
The chemical structure of **SirReal2** and **T1** is reported (**a**). Superimposition of all PDB IDs containing a **SirReal2**/SirReal2 analog available at the Protein Data Bank (PDB IDs: 4RMG, 4RMI, 4RMH, 5DY5, 5DY4, 5YQM, 5YQO, 5YQL, 5YQN). NAD^+^ is represented in red sticks, whereas the cartoon representation of the protein follows a gradient from blue to red (**b**). The SirReal2 binding mode (C atom; dark green) featured by the 4RMG complex is shown (**c**). NAD^+^ (by line) carbon atoms are colored in violet.

Due to the high incidence and severity of cancer, novel approaches to contrast tumor- progression and to overcome the resistance versus conventional anticancer therapeutics are required. The need for new strategies to treat cancer and the promising results of SIRT2 modulation on different types of cancers [7] steered the continuous research of effective SIRT2 modulators [20]. While SIRT2 activators have never been discovered, a number of SIRT2 inhibitors (SIRT2Is) have been identified in the past years: **AGK2 at** first [21] and then **SirReal2** (Figure 1a) later [22,23] are the most studied.

During the last years, several X-ray crystal structures of SIRT2 became available at the protein data bank [24,25] in the presence of different classes of enzyme inhibitors [26]. Regarding non-reaction mechanism-related SIRT2Is, most of the reported experimental data contain a SIRT2 isoform-selective ligand, with the SirReal2 analogs series being the most exploited. As previously reported by us [27,28], an abundance of X-ray SIRT2 structures complexes containing **SirReal2** or its derivatives is available (Figure 1b) [28].

As shown in Figure 1c, **SirReal2** efficiently bound SIRT2 featuring π–π stacking or van der Waals contacts between the terminal pyrimidine group and the residues Tyr139, Phe143, Ile169, or between the naphthyl groups and the residues Phe119, Phe131, Ile232, Phe234.

This piece of information allowed us to set up further structure-based studies to identify new putative SIRT2-targeting compounds aimed towards the discovery of several series of SIRT2Is via computational methods or structure–activity relationship expansion [28]. Recently, we reported computer-aided drug design methods leading to the identification of thiazole-containing derivatives exhibiting SIRT2 inhibitory ability and a higher selectivity for SIRT2 over SIRT6 [6], with the most effective being **T1** (SIRT2 IC_50_ = 17.3 μM) (Figure 1a). **T1** experienced protein−ligand interactions involving Phe96, Ile118, Phe119, and Ile232, as the reference SIRT2I **SirReal2**.

Herein, ligand-based studies have been performed to guide the rational design of novel thiazoles (**3a**–**3d**, **5a**, **5d**) as promising SIRT2Is, followed by molecular docking studies, chemical synthesis, and biological evaluation. Molecular docking results allowed us to highlight the ability of the new compounds to mimic the binding positioning of **SirReal2**, and the effectiveness of the proposed compounds **3a**–**3d** and **5a**, **5d** as novel SIRT2 inhibitors was confirmed by a subsequent in vitro evaluation.

## 2. Results and Discussion

### 2.1. Rational Design of Novel Thiazole-Containing SIRT2Is

During the last few years, some structure-based studies have been performed to guide the discovery of different series of SIRT2Is [28]. We reported in silico screening calculations leading to the discovery of thiazole derivatives (**T1**–**T6**; Figure S1) with sirtuin inhibitory ability [6]. Briefly, taking into account most of the available X-ray data for SIRT2, the majority of the SIRT2Is featured two aromatic terminal groups, with at least one of them being a heteroaromatic ring or exhibiting H-bonding moieties.

Most of the explored chemical diversity included thienopyrimidinone derivatives and SirReal2 congeners [28,29], bearing a monocyclic or a tricyclic main core tethered to quite flexible and aromatic terminal substituents. Both the two series of compounds achieved selectivity with respect to other sirtuins through induced-fit effects. The first ones featured a number of hydrophobic interactions with the biological target, including Phe235, Phe96, Ile232, Pro94, Pro140, Phe143, Leu206, Ile213, Phe214, Ile169, Leu138, Phe190 and Tyr139. In the case of thiazole compounds, such as SirReal2 analogs, their efficacy as SIRT2Is proved to be supported by hydrophobic contacts with Ile169, Leu134 and Leu138 and π–π stacking with Phe119.

On this basis, the in-house compounds thiazoles **T1**–**T6** have been validated as SIRT2Is via biological assays, matching the aforementioned ligand requirements [6]. Subsequent molecular docking analysis supported the whole study, revealing for **T1**, the most promising compound, the following features: (i) π–π stacking with Phe119, Phe234 and Phe235 and van der Waals contacts with Leu206 thanks to the terminal disubstituted phenyl ring; and (ii) further hydrophobic and π–π interactions between the other phenyl group and Leu106, Ile 213, Tyr139 and Phe143.

**Figure 2:**
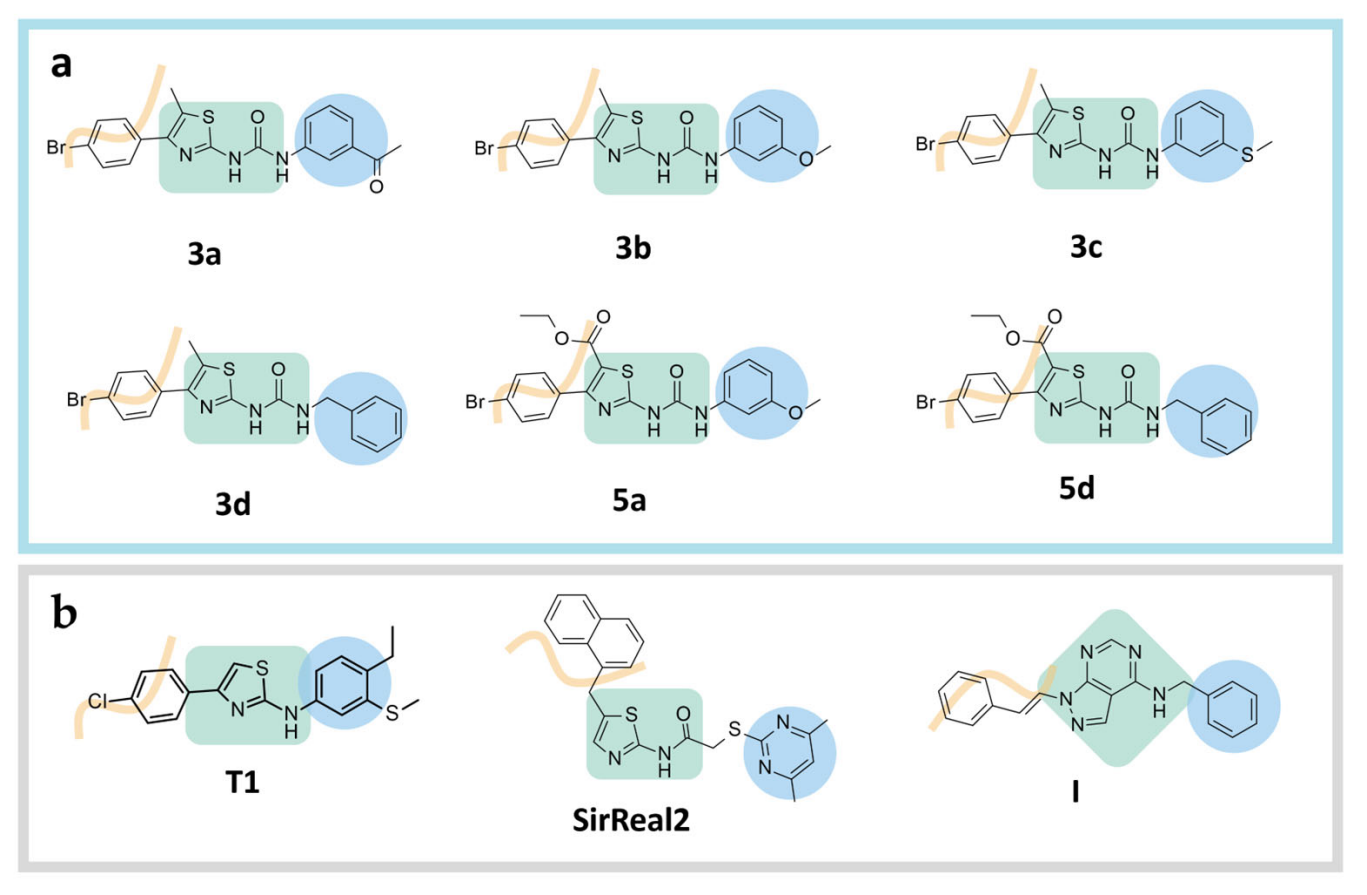
The chemical structure of the newly developed thiazole compounds as SIRT2Is is reported (**a**). The previous hit **T1** (in bold), the reference **SirReal2** and the pyrazole/pyrimidine inhibitor **I** are also shown (**b**). Comparable ligand features in terms of hydrophobic region, main central core and terminal aromatic group are highlighted in light orange, green, and cyan, respectively.

Herein, we proceeded with the in silico evaluation of a novel small series of thiazoles (**3a**–**3d**; **5a**, **5d**; Figure 2a), modified at the positions 2, 4 and 5 of the main thiazole core, with respect to the previous hits **T1**–**T6**.

The design of these derivatives has been obtained by merging key information from previously reported SIRT2Is. As shown in Figure 2b, three compounds have been considered: **SirReal2**, as a representative of its analogs, our reference hit **T1** (SIRT2 inhibition percentage = 100% at 150 μM) and a pyrazole/pyrimidine-based inhibitor (**I**; SIRT2 inhibition percentage = 81.2% at 150 μM). Our previous hit compound **T1** featured a central 2-amino thiazole core tethering two terminal aromatic groups, whereas both **SirReal2** and **I** displayed a bulkier linker (the 2-amido-thiazole core or the pyrazole-pyrimidine ring), as a corresponding bioisostere. As a consequence, the two aromatic substituents of **SirReal2** and **I** proved to be bulkier and more flexible than those experienced by **T1**. On the other hand, compound **I** suggested the effectiveness of a flat bicyclic ring in place of a monocyclic one when accompanied by at least one flexible aromatic pendant.

Thus, the ureide moiety in place of the previous **T1** amine group was attempted in **3a**–**3d** and **5a**, **5d** to mimic the role played by the conformationally locked thiazole-amide and pyrazole/pyrimidine core of **SirReal2** and **I**. The introduction of a further substituent at thiazole position 5 was investigated. In **3d** and **5d**, the presence of a benzyl substituent as a flexible terminal group was also explored.

To preliminary evaluate in silico the putative SIRT2 inhibitor ability of the novel thiazoles (**3a**–**3d**; **5a**, **5d**), a ligand-based approach has been applied [30]. A molecular interaction fields (MIFs) analysis has been performed, taking into account: (i) DRY to evaluate the role played by hydrophobic features; (ii) N1 to assess favorable interaction with the H-bond donor (HBD) probe; and (iii) O to assess favorable interaction with the H-bond acceptor (HBA) probe. Concomitant evaluation with respect to **SirReal2** of known SirReal2 analogs (**1**–**25**; chemical structure in Table S1) has been performed.

The results of each analysis include the calculation of each probe score, representing the degree of MIF overlap of the candidate molecule when compared with the reference compounds. The distance score indicates the overall difference in probe score evaluated between the investigated compounds and the template. The product of the individual probe scores gives the Glob_Prod global score, ranging from 0 (bad) to 1 (good) (details in Materials and Methods), which is considered for the compound ranking. For control compounds, the obtained results are shown in Table S2. Briefly, most of the effective SirReal2 analogs are properly top-ranked, as **14** (IC_50_ = 0.21 μM), whereas most of the less promising SIRT2Is have been classified as worst. In particular, three out of five of the most active SIRT2Is (IC_50_ values < 1.00 μM) fall in the three best-scored positions, with Glob-Prod values spanning from 0.6135 to 0.6632, whereas the predicted less promising **7**–**8** (IC_50_ = 143–207 μM) spanned from 0.4256 to 0.4999. On this basis, Glob-Prod values > 0.4000 were thought to be acceptable for the design of novel SIRT2Is, using **SirReal2** as a reference compound.

The comparison of MIF values on our novel compounds **3a**–**3d** and **5a**, **5d**, based on the reference **SirReal2**, highlighted five compounds (**3a**–**3d**; **5a**) characterized by Glob-Prod values of higher than 0.4000 (Table 1).

Among them, **3a**, **5a** and **3d** (Glob-Prod = 0.4362–0.4466) were predicted as most promising, with **3b**, **3c** (Glob-Prod = 0.4019–0.4082) also being mildly effective as putative SIRT2Is. On the contrary, **5d** was predicted as less potent than its analogs (Glob-Prod = 0.3678). An evaluation of the same MIF parameters with respect to compound **I** as a template revealed three compounds (**3a**, **3c**, **5a**) featuring Glob-Prod values higher than 0.4000 (Table 2).

These compounds featured Glob-Prod values spanning from 0.4003 to 0.4336. The analogs **3b**, **3d** and **5d** were predicted as less potent (Glob-Prod = 0.3845–0.3956). Interestingly, in both the two ligand-based analyses, compounds **3a** and **5a** were top-ranked. On the other hand, compounds **3c** and **3d** scored high in one of the two models.

**Figure 3:**
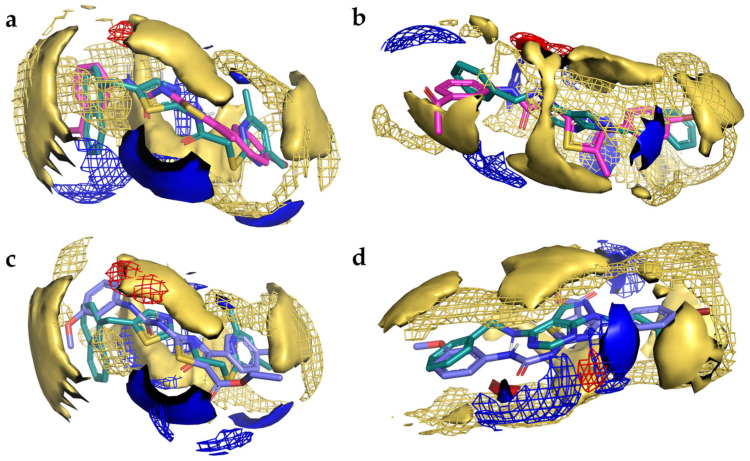
MIF Comparison of the template **SirReal2** (C atom; green) (**a**,**c**) and **I** (C atom; green) (**b**,**d**). MIFs (solid) and those (wireframes) of the candidates **3a** (C atom; magenta) (**a**,**b**) and **5a** (C atom; violet) (**c**,**d**) are reported. Blue: N1 MIF; red: O MIF; yellow: DRY MIF.

A perspective of MIFs comparison between **3a**, **5a** and the two references **SirReal2** and **I** is given in Figure 3. Favorable interactions with N1 MIF, O MIF and DRY MIF are reported in blue, red and yellow, respectively. This in turn suggested H-bond acceptor, H-bond donor and lipophilic or aromatic groups in the corresponding areas of the ligand.

**Figure 4:**
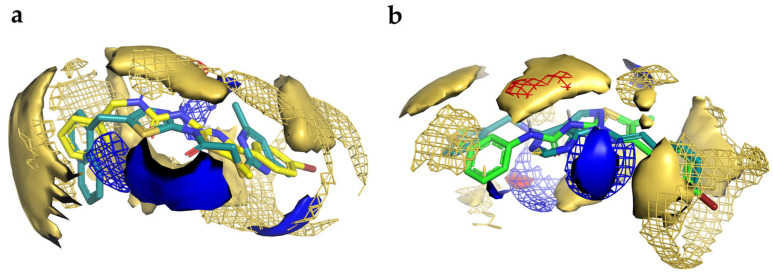
MIF Comparison of the template **SirReal2** (C atom; dark green) (**a**) and **I** (C atom; dark green) (**b**). MIFs (solid) and those (wireframes) of candidates **3d** (C atom; yellow) (**a**) and **3c** (C atom; green) (**b**) are reported. Blue: N1 MIF; red: O MIF; yellow: DRY MIF.

Compound **3a** has been predicted as properly matching most of the **SirReal2** and **I** DRY MIFs (Figure 3a,b; yellow areas), moving the acetamide phenyl ring and the terminal *p*-Br-phenyl one in the proximity of the naphthyl and pyrimidine ring of **SirReal2** and the amino benzyl and phenyl ring of **I**. With respect to **SirReal2**, the oxygen atom of the ureide moiety shared the same O MIF information represented around the reference compound oxygen atom (carbamide group). The H-bond donor ability of **3a** at the two ureide nitrogen atoms was in accordance with the N1 MIF featured by the reference **I** benzylamine group. The introduction in **5a** of the aromatic rings tethered to the central thiazole core has been confirmed to fulfill the requirements of the DRY MIF information by both references (**SirReal2**, Figure 3c; **I**, Figure 3d). In addition, the choice of the ester moiety at thiazole position 5 properly matched the DRY MIF and the O one, returning favored H-bonding groups at this position. Replacing the terminal phenyl ring tethered to the ureide moiety with more hydrophobic substituents, such as the benzyl group of **3d** or the 3-SCH3-phenyl ring of **3c**, was encouraged by DRY MIFs based on **SirReal2** (Figure 4a) and **I** (Figure 4b) being used as references.

In both analyses, the presence of a methyl group at thiazole position 5 was supported, in good agreement with the same MIF parameter.

Overall, ligand-based studies allowed us to compare the main steric and electrostatic features of **3a**–**3d** and **5a**, **5d** with those of **SirReal2** and **I**. Next, molecular docking calculations were exploited to assess the positioning of novel compounds.

### 2.2. Molecular Docking Studies

Molecular docking studies of compounds **3a**–**3d** and **5a**, **5d** have been performed, relying on the X-ray crystallographic structure of SIRT2 in the presence of **SirReal2** (PDB code = 4RMG) [22,23]. Details of the applied molecular docking approach are reported in the experimental section. The 4RMG co-crystallized ligand and analogs **1–25** have been re-docked in the crystal binding site to assess the reliability of the calculation and to interpret the final scoring function values (S values as the predicted ΔG value of each protein–ligand complex) [6]. The obtained values are shown in Table S3. Most of the SirReal2 analogs best-ranked conformers featured S values spanning from approximately −6.50 Kcal/mol, such as **21** (S = −6.58 Kcal/mol, SIRT2 IC_50_ = 502.8 μM), to −11.00 Kcal/mol, such as **14** (S = −10.994 Kcal/mol, SIRT2 IC_50_ = 0.21 μM), with the reference compound **SirReal2** (SIRT2 IC_50_ = 0.44 nM) having S = −9.55 Kcal/mol.

**Figure 5:**
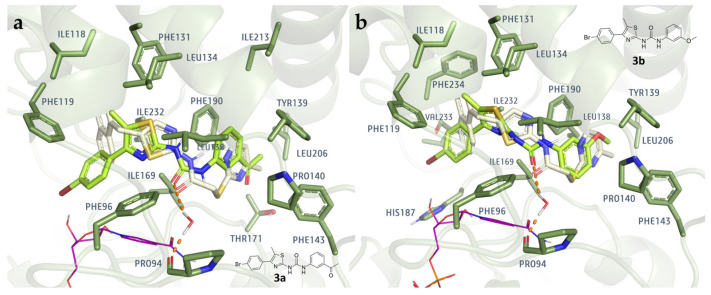
Docking positioning of compound **3a** (C atom, light green) (**a**) and **3b** (C atom; light green) (**b**) at the 4RMG binding site. The co-crystallized **SirReal2** (C atom; light yellow) structure is also reported. NAD^+^ (by line) carbon atoms are colored in violet.

Based on the PDB code 4RMG, **SirReal2** is bound at the interface between the zinc binding domain and the Rossmann fold and the area corresponding to the SIRT2 active site. An additional volume is created upon SirReal2 binding, resulting in a certain selectivity with respect to other SIRTs. This additional volume is called the “selectivity pocket” and is defined by residues Ile93, Ala135, Leu138, Pro140, Phe143, Leu206 and Ile213 (Figure 5).

The dimethyl pyrimidine moiety protrudes towards this area and establishes π–π-stacking interactions with Tyr139 and Phe190. The ligand carbonyl forms a water-mediated H-bond with the backbone of Pro94. Moreover, an intra-molecular H-bond is observed between one N of the pyrimidine and the amidic NH; this interaction rigidifies the ligand. The thiazole moiety is located near Leu138, Leu134 and Ile169. Residues Ile118, Phe234, Phe119, Phe131, Leu134, Ile232 and Ile169 establish non-polar contacts with the ligand naphthyl group.

Regarding the novel thiazoles **3a**–**3d** and **5a**, **5d** compounds, the final scoring functions are listed in Table S4, with S values spanning from approximately −8.30 Kcal/mol, such as **3b** (S = −8.30 Kcal/mol), to −10.50 Kcal/mol, such as **3c** (S = −10.54 Kcal/mol). Thus, all of the proposed novel thiazoles have been retained as putative SIRT2Is, featuring best-scored S values comparable with those exhibited by most of the SirReal2 analogs, **1–25**. Accordingly, all of them have been analyzed by visual inspection.

Among the phenyl-substituted ureide containing thiazoles **3a**–**3c** and **5a**, compounds **3a** and **3b** shared the same substituents at thiazole position 5, whereas the ureide-substituted phenyl ring displayed H-bonding and polar groups such as the acetamide (**3a**) and the methoxy group (**3b**). As shown in Figure 5a, the acetamide phenyl substituent of **3a** established π–π stacking with Phe190 and Tyr139, whereas the acetyl group points towards Leu206. This group also contacts Pro140 and Thr171. The ureide moiety mimics the amide of **SirReal2** (light yellow) as it establishes a water-mediated H-bond with Pro94. The methyl group of **3a** displayed van der Waals contacts with Leu134, Ile169, Phe96, Leu138 and Ile232, and the aromatic group at thiazole position 4 interacts with Phe119 and Ile169. Analog **3b** experienced a similar docking mode, and the methoxy-substituted aromatic ring exhibits an offset π-stacking with Phe190 and a non-polar interaction with Pro140, Leu206 and Phe143. The ureide moiety forms a water-mediated H-bond with Pro94 through its carbonyl group. The phenyl ring at thiazole position 4 and the thiazole core interact with the surrounding hydrophobic residues Ile118, Phe119, Phe96, Phe234, Leu134, Ile196 and Ile232.

**Figure 6:**
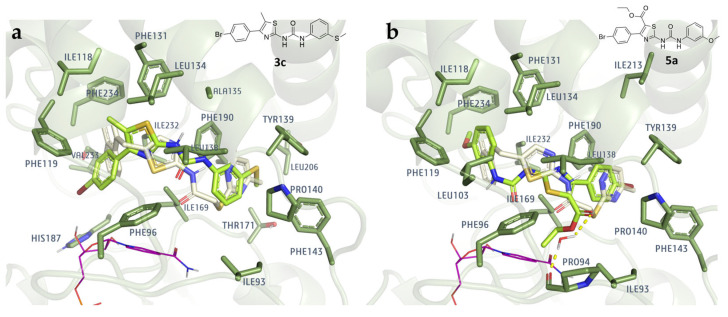
Docking positioning of compound **3c** (C atom, yellow) (**a**) and **5a** (C atom; yellow) (**b**) at the 4RMG binding site. The co-crystallized **SirReal2** (C atom; dark green) structure is also reported. NAD^+^ (by line) carbon atoms are colored in violet.

Removing the acetamide and methoxy substituents by the ureide phenyl portion of the ligand, in favor of the thiomethyl moiety (**3c**), allowed us to maintain a comparable docking positioning (Figure 6a).

The thiazole **3c** moved the thiomethyl phenyl group towards Phe190, also featuring a non-polar interaction with Pro140. The thiomethyl substituent displayed more van der Waals contacts than **3a** and **3b** with Leu206, Tyr139, Phe143 and Thr171. The ureide moiety facing residue Leu138 is also involved in a water-mediated H-bond with Pro94. The aromatic group at thiazole position 4 is engaged in the same pattern of contacts as **3a**, **3b**.

Maintaining a polar group at the ureide-containing phenyl moiety with the replacement of the methyl substituent at thiazole position 5 with a bulkier hydrophobic group, such as the ester moiety (**5a**), improved the number of van der Waals contacts with the enzyme. As shown in Figure 6b, the phenyl ring at thiazole position 4 of **5a** establishes π–π interactions with Phe190 and Tyr139 and van der Waals contacts with Pro140. The central thiazole ring is inserted in a hydrophobic environment formed by residues Phe190, Leu138, Ile169 and Phe96. In this case, the bulky ester moiety protrudes towards the nicotinamide group of NAD^+^, while the carbonyl oxygen atom is engaged in the water-mediated H-bond with Pro94. The ureide-substituted thiazole core simulates the positioning of the **SirReal2** naphthyl-based thiazole moiety; π–π stacking and hydrophobic contacts with Phe119, Phe131 and Ile232, Ile118, Phe234, Leu134 have been maintained.

**Figure 7:**
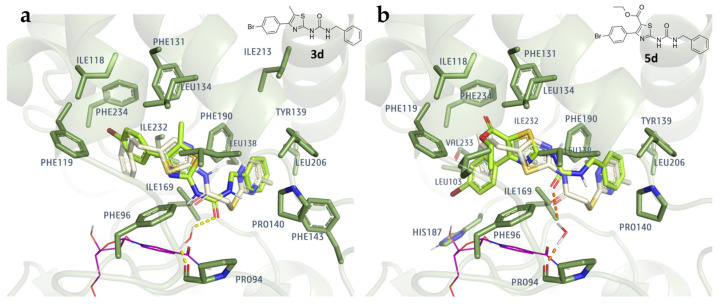
Docking positioning of compound **3d** (C atom, light green) (**a**) and **5d** (C atom; light green) (**b**) at the 4RMG binding site. The co-crystallized **SirReal2** (C atom; light yellow) structure is also reported. NAD^+^ (by line) carbon atoms are colored in violet.

The introduction of the ureide-based benzyl group in place of the ureide-based phenyl one led to compounds **3d** and **5d** (Figure 7).

Based on our calculation, **3d** fulfilled the pocket steric and electrostatic properties better than **5d**, bearing a smaller substituent at thiazole position 5. In any case, the required polar contact with Pro94 was displayed. For **3d** (Figure 7a), the *p*-Br-phenyl group was quite superposed to the reference inhibitor naphthyl group, with the small methyl one being projected towards Leu134. Conversely, the bulkier ester moiety of **5d** at the same position moved the compound out of the binding pocket in comparison with **SirReal2** (Figure 7b).

### 2.3. Chemical Synthesis

**Scheme 1:**
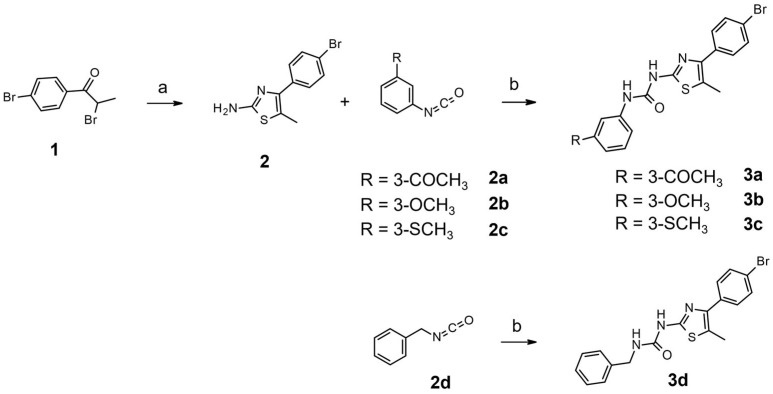
Reagents and conditions: (**a**) thiourea, ethanol, reflux, 6 h; (**b**) anhydrous acetonitrile, 80 °C for 3 h then room temperature for 1 h.

**Scheme 2:**
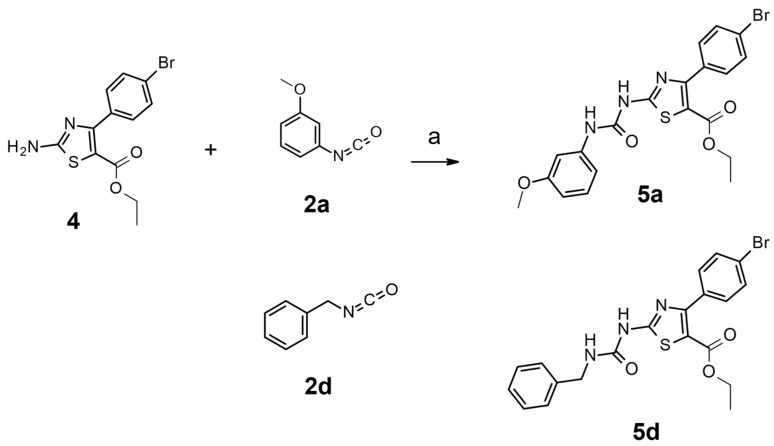
Reagents and conditions: (**a**) anhydrous acetonitrile, 80 °C for 6 h.

Urea analogs **3a**–**3d** and **5a, 5d** were prepared in fairly good yields (in a range from 27% to 45%) by coupling the aminothiazole derivative **2**, obtained by coupling suitable 2-bromopropiophenone **1** with thiourea, or commercially available as **4** with substituted phenyl isocyanates **2a**–**2d** in dry acetonitrile at T = 80° (Scheme 1 and Scheme 2).

Specifically, the aminothiazole moiety **2** was synthetized using the reaction of suitable haloketone and thiourea, following the general Hantzsch cyclocondensation pattern according to a protocol previously published by us [31]. Instead, for the synthesis of final urea analogs, we adapted a procedure previously published with appropriate modifications [32]. All molecules synthetized have been characterized with HPLC-MS and NMR analyses.

### 2.4. Biochemistry and Biological Assays

#### 2.4.1. Biological Evaluation of Urea Derivatives as SIRT2 Inhibitors

The effect of urea derivatives on SIRT2 deacetylase activity was evaluated upon incubation of recombinant SIRT2 with an acetylated peptide (H3K9Ac, a peptide acetylated on Lys 9) and the co-substrate NAD^+^. The percentage of SIRT2 activity inhibition is presented in Table 3.

We subsequently determined the IC_50_ values for the compounds inhibiting >95% of the activity when added at 150 µM: all compounds were found to inhibit SIRT2 activity in the micromolar range, with **5a** having the lowest IC_50_ (Table 3).

Next, the selectivity of the compounds for SIRT2 against other sirtuins was also determined. The compounds were tested at the final concentration of 150 µM in the deacetylation reaction of SIRT1, SIRT3 and SIRT6, with this last one being promoted by the addition of the SIRT6 activator **MDL-800** and also in the depalmitoylation reaction of SIRT6. The % inhibition obtained in the presence of each compound is reported in Table 4.

Interestingly, all of the newly developed compounds featured dual SIRT1/2 inhibitory ability, being selective over SIRT6. The introduction of the ureide moiety is thought to be advantageous for the design of dual acting compounds, being comparable with the thioureide and ureide chemotypes previously exploited in the search of SIRT1 modulators [28,33,34,35]. On the other hand, the choice of the thiazole core allowed selectivity towards the SIRT6 enzyme to be maintained, as previously reported for **T1**–**T6** and for the reference compound **SirReal2** [6].

Given that compound **5a** exhibited the lowest IC_50_ value and the highest selectivity over SIRT6, the following experiments on cells were performed using this compound.

#### 2.4.2. SIRT2 Expression Steadily Increases at Different Stages of Skin Carcinogenesis

**Figure 8:**
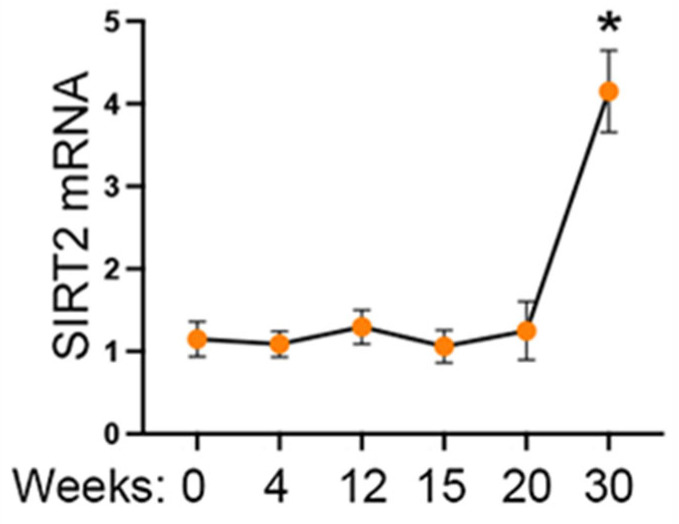
SIRT2 expression during skin carcinogenesis in a DMBA-TPA mouse model. SIRT2 mRNA levels in the DS of mice in healthy skin (0 weeks) and at different stages of skin carcinogenesis in DMBA-TPA-treated D2^3xflag^ mice were investigated by RT-PCR analysis (*n* = 6). * *p* < 0.05.

Recently, we demonstrated that SIRT6 inhibition delays skin cancer progression in cutaneous squamous cell carcinoma (cSCC) [36]. SIRT2 has been reported to be overexpressed at both the mRNA and the protein levels in human cSCC tissues [36]. SIRT2 expression was analyzed in dorsal skin (DS) tissues collected from DMBA-TPA-treated mice at different stages of carcinogenesis. SIRT2 mRNA started to increase at the very late stage of multistep chemical carcinogenesis. Indeed, we observed a drastic increase following 20 weeks of DMBA-TPA treatment, when papilloma stage lesions undergo conversion into SCCs (30 weeks from DMBA treatment) (Figure 8). This result confirms the observations of Jacobson’s group, suggesting an oncopromoter role of this sirtuin in cSCC and, more precisely, indicating that SIRT2 can drive the progression of skin tumors toward the most advanced and invasive forms. In turn, these data per se suggest that SIRT2 inhibition, beside SIRT6 inhibition, may represent a promising strategy to treat advanced cSCC.

#### 2.4.3. Cell Toxicity on SCC13 Cell and Inhibition of Endogenous SIRT2

**Figure 9:**
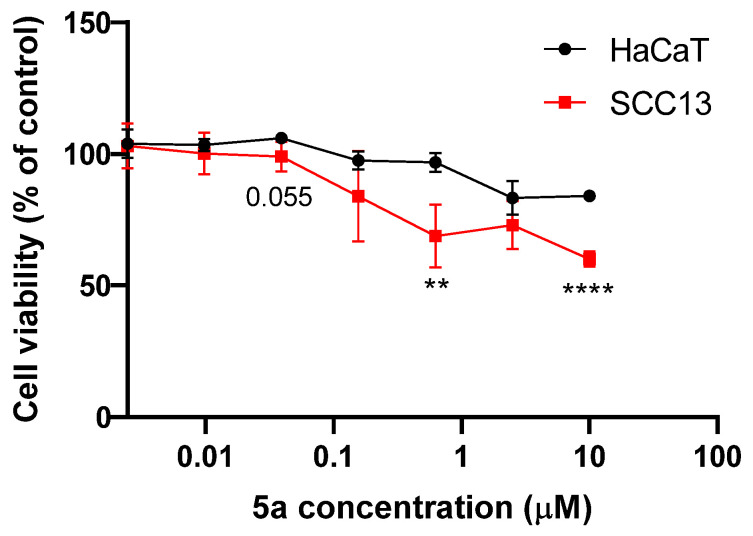
Compound **5a** shows a concentration-dependent toxicity on SCC13, but not on the normal keratinocyte cell line HaCaT. Cells were incubated for 48 h with increasing concentrations of **5a**. Cell viability was measured by an SRB assay. The graph represents the mean ± SD of 3 independent experiments. ** *p* < 0.01, **** *p* < 0.0001.

Treatment of SCC13 cells with increasing concentrations of **5a** determined a concentration-dependent cell death, as measured after 48 h (Figure 9). This cell mortality is consistent with the IC_50_ of the compound (8.6 μM) on the recombinant protein. Moreover, this result, together with the effect of **5a** on the acetylation of α-tubulin (see the Western blot analysis below) confirms that **5a** can enter cells and inhibit SIRT2.

**Figure 10:**
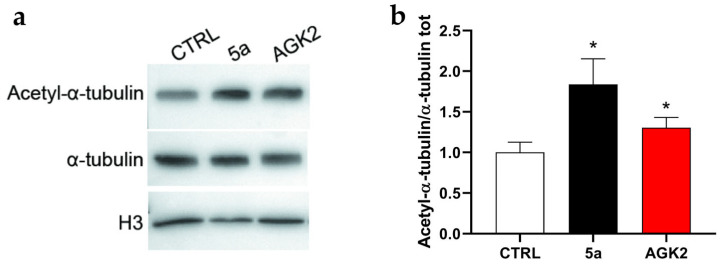
Compound **5a** increases α-tubulin acetylation in SCC13 cells. Cells were incubated with 5 μM compound **5a** or AGK2 or the respective amount of vehicle DMSO and used for protein lysate generation. Total, acetylated α-tubulin and H3 histone levels were detected by immunoblotting (**a**), quantified, normalized to total α-tubulin and expressed as percentage increase vs. vehicle-treated cells (**b**). One representative Western blot analysis and the mean ± SD of 3 quantifications is shown. * *p* < 0.05.

To confirm that the effect of **5a** on cell viability was due to its ability to inhibit the endogenous SIRT2, Western blot analyses were performed to quantify the level of acetylation of α-tubulin, a direct substrate of SIRT2 enzymatic activity. SCC13 cells treated with 5 μM **5a** or with AGK2 (used as positive control) for 48 h exhibited increased levels of alpha-tubulin acetylation compared with control cells (Figure 10).

Our results show that SIRT2 expression increases during skin carcinogenesis from the early stages to reach its maximal expression in the SCC stage. These results align with the observation of Jacobson’s group in actinic keratoses and SCC cells [37], suggesting an oncopromoter role of SIRT2. Nevertheless, as for other sirtuins, the role of SIRT2 is also controversial. Other studies suggest an oncosuppressive role of SIRT2 in SCC due to its epigenetic induction of skin cell differentiation [36,38]. Possibly, as reported for the other sirtuins [39,40], the role of SIRT2 as an oncosuppressor or oncopromoter gene depends on the stage of the disease and on the cell type analyzed. For this reason, the treatment of SCC or other malignancies with pharmacological agents targeting sirtuins should always be preceded by analyses to define the most appropriate tumor stage for the therapeutic intervention towards a more personalized approach.

## 3. Materials and Methods

### 3.1. Molecular Modeling Studies

All the studied derivatives were built in silico by the MOE Builder program included in MOE 2019.01 software [41] and assigned the prevalent protonation state at pH = 7.4 by means of the wash function. Ligands have been parametrized with the AM1 partial charges calculation method. The energy minimization step has been performed using the Energy Minimize tool using an MMFF94x forcefield, as implemented in MOE. The RMS (root mean square) gradient was equal to 0.0001, with the root mean square gradient being the norm of the gradient times the square root of the number of (unfixed) atoms.

The ligand-based studies were developed with the FLAP2.2.1 Ligand-Based module [42,43], relying on the X-crystallographic structure of **SirReal2** (PDB code = 4RMG) [22] and on the previously derived docking pose of **I [6]** as reference templates.

For each of the candidates, 25 conformers were generated starting from their optimized geometry (compound preparation step previously described). Four probes were chosen for the ligand-based analysis, namely the H (expressing shape similarity parameter), DRY (hydrophobic parameter), O (HBA probe, corresponding to a carbonyl-group) and N1 probes (HBD probe, corresponding to amide NH). Details of the calculations have been previously described by us [30]. The probe scores represent the degree of overlap of the MIFs of the evaluated molecule with respect to the template for each probe individually. The distance score represents the overall difference according to the probe score between the ligand and reference compound. MIF information was exploited to visually compare the newly designed molecules and the known SIRT2Is **SirReal2** and **I**. Indeed, the superposition of the same kind of MIF between the novel compound and the corresponding template is regarded as a sign of ligand similarity, enhancing the probability of featuring a similar biological ability [42].

All the molecular docking simulations at the 4RMG protein have been performed by means of the DOCK tool included in MOE software via template-based approach using the previously described protocol [6,44,45]. All those residues placed at 4.5 Å distance from the PDB code co-crystallized inhibitor have been considered.

The alpha triangle method and Affinity ΔG prediction as the final scoring function have been applied to obtain the 50 to 10 best-scored conformers. Details of the template-based docking studies and of the available scoring functions are reported in the literature [46,47].

### 3.2. Chemistry

All solvents and chemicals were of reagent grade. Unless otherwise mentioned, all solvents and chemicals were acquired from Sigma Aldrich (St. Louis, MO, USA), VWR (Radnor Township, PA, USA).

The analytical instrument used was an Agilent 1260 high-performance liquid chromatograph (HPLC, Agilent Technologies, Santa Clara, CA, USA). The analytical HPLC column was a Phenomenex column (Torrance, CA, USA) C18 Luna (4.6 × 250 mm, 5 µm).

The preparative HPLC was an Agilent 1260 Infinity preparative HPLC, and a Phenomenex C18 Luna (21.2 × 250 mm, 15 µm) column was used for preparative chromatography.

Liquid chromatography–electrospray mass spectrometry (HPLC-ESI-MS, Agilent Technologies, Santa Clara, CA, USA) was used to analyze the intermediates and the raw products using an Agilent 1100 series LC/MSD ion trap instrument.

The HRMS experiments were performed using a Q Exactive Orbitrap instrument by Thermo Scientific (Waltham, MA, USA).

The nuclear magnetic resonance (NMR) analyses were performed using a Jeol 400 MHz spectrometer (Jeol LTD, Akishima, Tokyo, Japan).

The proton spectra and the carbon spectra were acquired at room temperature at 400 MHz and at 100 MHz, respectively. Chemical shifts were reported in units (ppm) relative to TMS as an internal standard. Coupling constants (J) were reported in Hertz (Hz).

All raw products were purified with preparative HPLC using the following gradient: from 0 to 5 min at 20% eluent B, from 5 min to 40 min at 100% eluent B and from 40 to 45 min at 100% eluent B. Eluent A was water with 0.1% formic acid (FOA), and eluent B was acetonitrile with 0.1% FOA. All final products utilized in biological assays had a purity of 95% or higher based on the analytical HPLC/MS analysis.

Compound purity was determined by integrating peak areas of the chromatogram obtained in liquid phase, monitored at 254 nm.

1-(3-acetylphenyl)-3-(4-(4-bromophenyl)-5-methylthiazol-2-yl)urea **3a**

To a solution of commercially available 2,4′-dibromopropiophenone (292 mg, 1 mmol) in ethanol absolute (2 mL), we added thiourea (76 mg, 1 mmol), and the reaction was heated to reflux for 6 h. The mixture was then extracted with diethyl ether (3 × 3 mL) and washed with water (2 × 1 mL). The organic phase was dried under vacuum. The product was then purified by preparative HPLC to afford 4-(4-bromophenyl)-5-methylthiazol-2-amine (224 mg, 83%) ESI-MS: *m*/*z* 270.1 [M+H]^+^.

To 4-(4-bromophenyl)-5-methylthiazol-2-amine (54 mg, 0.2 mmol) in dry acetonitrile, we added 3-acetylphenyl isocyanate (32 mg,0.2 mmol) and stirred at T = 80 °C for 3 h and then at room temperature for 1 h. A white solid precipitated from the reaction mixture, which was collected by centrifugation. The dry solid was recrystallized from absolute ethanol to obtain the final product (38 mg, 44%).

The HRMS (ESI) calculated for C_19_H_17_BrN_3_O_2_S [M+H]^+^ 430.02248 found 430.02193

^1^H NMR (400 MHz, DMSO-d6): δ 9.76 (s, NH, 1H); 7.73–7.52 (m, 4H, arom); 7.27–7.14 (m, 2H, arom), 6.87 (dd, J = 7.2, 2.0, 1H, arom); 6.71 (dd, J = 2.0, 1.2, 1H, arom); 2.52 (s, 3H, CH_3_CO); 2.43 (3, 3H, CH_3_).

^13^C NMR (100 MHz, DMSO-d6): δ 197.78, 156.66, 154.70, 143.68, 139.98, 138.51, 133.97, 130.92, 130.14, 128.63, 121.73, 120.97, 120.38, 115.32, 28.42, 12.49.

1-(4-(4-bromophenyl)-5-methylthiazol-2-yl)-3-(3-methoxyphenyl)urea **3b**

Compound **3b** (30 mg, 36%) was prepared from 4-(4-bromophenyl)-5-methylthiazol-2-amine (54 mg, 0.2 mmol) and 3-methoxyphenyl isocyanate (30 mg, 0.2 mmol) in the same manner as described for **3a** as a white solid.

The HRMS (ESI) calculated for C_18_H_17_BrN_3_O_2_S [M+H]^+^ 418.02248 found 418.02185

^1^H NMR (400 MHz, DMSO-d6): δ 9.52 (s, NH, 1H); 7.74–7.52 (m, 4H, arom); 7.28–7.15 (m, 2H, arom), 6.94 (dd, J = 8.0, 2.0, 1H, arom); 6.66 (dd, J = 2.4, 1.2, 1H, arom); 3.73 (s, 3H, OCH_3_); 2.43 (3, 3H, CH_3_).

^13^C NMR (100 MHz, DMSO-d6): δ 156.35, 154.37, 142.16, 140.65, 138.98, 134.62, 131.63, 129.58, 128.21, 121.48, 120.74, 118.43, 115.44, 105.84, 55.52, 12.47.

1-(4-(4-bromophenyl)-5-methylthiazol-2-yl)-3-(3-(methylthio)phenyl)urea **3c**

Compound **3c** (39 mg, 45%) was prepared from 4-(4-bromophenyl)-5-methylthiazol-2-amine (54 mg, 0.2 mmol) and 3-(Methylthio)phenyl isocyanate (33 mg, 0.2 mmol) in the same manner as described for **3a** as a grey solid.

The HRMS (ESI) calculated for C_18_H_17_BrN_3_OS_2_ [M+H]^+^ 433.99963 found 433.99901

^1^H NMR (400 MHz, DMSO-d6): δ 9.86 (s, NH, 1H); 7.63–7.52 (m, 4H, arom); 7.44 (m, 1H, arom); 7.25–7.11 (m, 2H, arom), 6.88 (dd, J = 7.6, 1.6, 1H, arom); 2.43 (s, 3H, CH_3_); 2.41 (s, 3H, SCH_3_).

^13^C NMR (100 MHz, DMSO-d6): δ 155.60, 152.23, 143.04, 139.91, 139.37, 134.62, 131.82, 130.45, 129.96, 121.45, 120.84, 120.42, 115.80, 115.42, 15.15, 12.43.

1-benzyl-3-(4-(4-bromophenyl)-5-methylthiazol-2-yl)urea **3d**

Compound **3d** (33 mg, 41%) was prepared from 4-(4-bromophenyl)-5-methylthiazol-2-amine (54 mg, 0.2 mmol) and benzyl isocyanate (26.5 mg, 0.2 mmol) in the same manner as described for **3a** as a pale grey solid.

The HRMS (ESI) calculated for C_18_H_17_BrN_3_OS [M+H]^+^ 402.02756 found 402.02714.

^1^H NMR (400 MHz, DMSO-d6): δ 9.42 (s, NH, 1H); 7.78–7.46 (m, 5H, arom); 7.35–7.18 (m, 4H, arom); 4.23 (s, 2H, CH_2_N); 4.15 (q, J = 7.6, 2H, OCH_2_); 1.18 (t, J = 7.6, 3H, CH_3_CH_2_)

^13^C NMR (100 MHz, DMSO-d6): δ 156.69, 153.14, 144.78, 139.99, 139.06, 134.59, 131.75, 130.47, 128.72, 123.44, 122.95, 121.12, 117.82, 112.70, 44.94, 12.52.

Ethyl4-(4-bromophenyl)-2-(3-(3-methoxyphenyl)ureido)thiazole-5 carboxylate **5a**

To ethyl 2-amino-4-(4-bromophenyl) thiazole-5-carboxylate (65 mg, 0.1 mmol) in dry acetonitrile, we added 3-methoxyphenyl isocyanate (30 mg, 0.2 mmol) dropwise and stirred at T = 80 °C for 6 h. Then, the mixture was cooled to room temperature and the colorless crystalline precipitate dissolved in dichloromethane (3 mL) and washed with H_2_O (3 × 2 mL). The organic phase was dried over Na_2_SO_4_, concentrated under reduced pressure and purified by preparative HPLC. The peak of interest was concentrated to obtain the title compound (30.5 mg, 32%).

The HRMS (ESI) calculated for C_20_H_19_BrN_3_O_4_S [M+H]^+^ 476.02795 found 476.02755

^1^H NMR (400 MHz, DMSO-d6): δ 9.09 (s, NH, 1H); 7.71–7.53 (m, 4H, arom); 7.23–7.12 (m, 2H, arom), 6.95 (dd, J = 8.0, 2.0, 1H, arom); 6.61 (dd, J = 2.5, 1.0 1H, arom); 4.15 (q, J = 7.2, 2H, OCH_2_); 3.71 (s, 3H, OCH_3_); 1.18 (t, J = 7.2, 3H, CH_3_CH_2_)

^13^C NMR (100 MHz, DMSO-d6): δ 161.80, 160.26, 139.99, 133.81, 132.32, 131.13, 130.31, 122.93, 111.62, 109.15, 105.01, 61.27, 55.59, 29.57, 14.55,

Ethyl 2-(3-benzylureido)-4-(4-bromophenyl)thiazole-5-carboxylate **5d**

To ethyl 2-amino-4-(4-bromophenyl) thiazole-5-carboxylate (65 mg, 0.1 mmol) in dry acetonitrile, we added benzyl isocyanate (26.5 mg,0.2 mmol) dropwise and stirred at T = 80 °C for 8 h. Then, the mixture was cooled to room temperature and concentrated under vacuum and after extraction with ethyl acetate (3 × 3 mL), washed with H_2_O (3 × 2 mL). The organic phase was dried over Na_2_SO_4_, concentrated under reduced pressure and purified by preparative HPLC. The peak of interest was concentrated to obtain the title compound (25 mg, 27%).

The HRMS (ESI) calculated for C_20_H_19_BrN_3_O_3_S[M+H]^+^ 460.03304 found 460.03260

^1^H NMR (400 MHz, DMSO-d6): δ 9.27 (s, NH, 1H); 7.79–7.44 (m, 5H, arom); 7.36–7.21 (m, 4H, arom); 4.26 (s, 2H, CH_2_N); 4.13 (q, J = 7.6, 2H, OCH_2_); 1.19 (t, J = 7.6, 3H, CH3CH2)

^13^C NMR (100 MHz, DMSO-d6): δ 161.82, 160.37, 147.06, 139.91, 139.17, 133.52, 131.19, 130.48, 128.88, 123.46, 122.97, 121.08, 112.71, 61.26, 45.53, 14.55.

### 3.3. Enzymatic Assays

The synthesis of peptides H3K9Ac and H3K9Palm (Acetylated and Palmitoylated, respectively) was carried out as in [27]. The evaluation of SIRT2, SIRT1, SIRT3 and SIRT6 deacetylase activity and SIRT6 depalmitoylase activity were performed following the procedures described in [27].

### 3.4. Cell Culture and Compound Toxicity Assay

SCC13 and HaCaT cell lines were a kind gift from Prof. Alessio Nencioni (University of Genoa), and cells were maintained in Gibco™ Keratinocyte SFM 1X medium supplied with prequalified human recombinant epidermal growth factor 1–53 (EGF 1–53) and bovine pituitary extract (BPE, CAT# 17005042) (Thermo Fisher Scientific, Milano, Italy), with 50 IU/mL penicillin and 50 μg/mL streptomycin and cultured as in [36]. SCC13 and HaCaT cells were treated for 48 h with the putative SIRT2 inhibitor, and cell viability was analyzed by a sulforhodamine B assay. Briefly, 5000 cells/well were seeded in 96-well plates in triplicate and allowed to adhere overnight. The day after, the medium was replaced with fresh medium containing increasing concentrations of **5a**. After 48 h, TCA was added, and samples were processed as previously described [48].

### 3.5. Western Blotting

SCC13 cells (3 × 10^5^) were seeded in 60 mm Petri dishes and left to adhere overnight. The day after, the medium was replaced with a medium containing **5a** at a final concentration of 5 µM or the respective amount of DMSO vehicle. After 48 h, the medium was removed, and cells were washed with PBS and lysed by scraping with 100 µL of lysis buffer (25 mM Tris-HCl, pH 7.8; 2 mM DTT; 2 mM EDTA, 10% glycerol; 1% Triton^®^ X-100). The lysate was clarified by centrifugation, and the protein concentration was measured using the Bradford assay. Twenty µg of proteins were loaded on a 10% polyacrylamide gel and then transferred to a nitrocellulose membrane. The membrane was saturated with 5% skim milk powder solution in TBS-Tween 0.05%. To determine the acetylation of alpha-tubulin, membranes were incubated with an anti-acetyl-alpha-tubulin antibody (#5335, Cell Signaling Technology, Danvers, MA, USA), whose signal was normalized on the total amount of alpha-tubulin (#2125, Cell Signaling Technology).

### 3.6. Cutaneous Chemical Carcinogenesis in Animals

The generation of D2^3xflag^ mice and the DMBA-TPA treatment inducing cutaneous chemical carcinogenesis have already been described [36,49]. All animal experiments were carried out in the animal facility of CEINGE-Biotecnologie Avanzate, Napoli, Italy, following ethical institutional guidelines. Dorsal skin samples were taken at different time points: mRNA extraction, retrotranscription, and qPCR analyses were performed as previously described [50] using the following primers: cyclophilin A (*CypA*), forward 5′-CGCCACTGTCGCTTTTCG-3′, reverse 5′-AACTTTGTCTGCAAACAGCTC-3′; sirtuin 2 (Sirt2), forward 5′-TGCAGGAGGCTCAGGATTCA-3′ and reverse 5′-AGACGCTCCTTTTGGGAACC-3′.

## 4. Conclusions

The rational design of SIRTIs is gaining attention for the treatment of different cancer types but also to contrast neurodegenerative disease, diabetes and autoimmune syndromes. Recently, a number of structure-based studies have been attempted to explore the potency and selectivity of different series of SIRT2Is. Our previously developed structure-based studies allowed us to discover thiazole-containing derivatives as selective SIRT2Is, such as **T1**. Related molecular docking studies revealed key contacts for this hit compound: π–π stacking with Phe119, Phe234 and Phe235 and van der Waals contacts with Leu206, Leu106, Ile 213, Tyr139 and Phe143. This knowledge has been exploited in tandem with ligand-based analyses to investigate in silico the putative SIRT2 inhibitory ability of the herein developed **3a**–**3d** and **5a**, **5d**, designed with the introduction of a substituent at thiazole position 5 and a ureide moiety in place of the previous **T1** amine group.

To preliminarily evaluate these compounds in silico as putative SIRT2 inhibitors, a ligand-based approach has been applied, comparing their MIFs with those of reference inhibitors (**SirReal2** and the pyrazole/pyrimidine compound **I**). In both analyses, compounds **3a** and **5a** were predicted as the most promising. In addition, based on the MIFs evaluation with respect to **SirReal2** and **I**, compound **3d** and **3c** were also evaluated as putative SIRT2Is. The following molecular docking studies showed compound **3a** as the most promising 5-CH_3_-4-phenyl-substituted thiazoles herein investigated. The compounds established π–π stacking with Phe190 and Tyr139 and hydrophobic contacts with Leu206. Notably, the **3a** ureide moiety mimics the amide of **SirReal2**, featuring a water-mediated H-bond with Pro94. The replacement of the methyl substituent in favor of the ester moiety, as shown by **5a**, was predicted as beneficial, leading to increased van der Waals contacts with the enzyme. Then, the introduction of the ureide-based benzyl group (**3d** and **5d**) in place of the ureide-based phenyl one was evaluated as advantageous. Compound **3d** was predicted to be much more promising than **5d**. Indeed, the **3d** *p*-Br-phenyl group mimics the **SirReal2** naphthyl group, with the small methyl substituent being projected towards Leu134. Subsequent biological assays confirmed compounds **3a** (SIRT2 IC_50_ = 37 µM) and **5a** (SIRT2 IC_50_ = 9 µM) as SIRT2Is. In vitro assays pointed to **5a** as a promising anticancer agent to be optimized for the treatment of SCC and other malignancies where SIRT2 plays a prominent role in cancer development and progression. The use of SIRT2Is to treat malignances and other pathologies should face the controversial role of SIRT2; thus, careful analysis should be taken case by case following the pathway of a personalized therapy.

## Figures and Tables

**Table 1:** The calculated scoring function Glob-Prod values based on the MIFs H, N1, DRY and O are reported (compounds **3a**–**3d** and **5a**, **5d** as candidates, with **SirReal2** the template). The most promising compounds, **3d**, **5a**, **3a**, are depicted in green. Top-ranked compounds in both models are underlined.

Candidate	Glob-Prod	H	N1	DRY	O
**3d**	0.4466	0.5974	0.3394	0.2161	0.5252
**5a**	0.4395	0.5478	0.2923	0.2359	0.5170
**3a**	0.4362	0.5559	0.3251	0.2322	0.6322
**3b**	0.4082	0.6055	0.2650	0.2082	0.4809
**3c**	0.4019	0.5713	0.3339	0.2203	0.4606
**5d**	0.3678	0.5328	0.2794	0.1907	0.4949

**Table 2:** The calculated scoring function Glob-Prod values based on the MIFs H, N1, DRY and O are reported (compounds **3a**–**3d** and **5a**, **5d** as candidates, with **I** being the template). The most promising compounds, **3a**, **5a**, **3c**, are depicted in green. Top-ranked compounds in both models are underlined.

Candidate	Glob-Prod	H	N1	DRY	O
**3a**	0.4336	0.5675	0.3390	0.2721	0.5493
**5a**	0.4309	0.5426	0.3135	0.2537	0.4184
**3c**	0.4003	0.6403	0.3017	0.2784	0.5173
**3b**	0.3956	0.5866	0.2632	0.2707	0.5393
**3d**	0.3858	0.6557	0.2791	0.2662	0.4844
**5d**	0.3845	0.6453	0.2382	0.3388	0.4662

**Table 3:** Compounds activity as putative inhibitors of SIRT2, tested on the recombinant protein.

Compound	% Inhibition SIRT2 (at 150 μM)	SIRT2 IC_50_ (μM)
**3a**	100 ± 5	37 ± 2
**3d**	100 ± 4	108 ± 4
**5a**	98 ± 5	9 ± 2
**5d**	93 ± 4	n.d.
**3b**	93 ± 4	n.d.
**3c**	96 ± 6	26 ± 4

n.d., not determined.

**Table 4:** Compounds activity as inhibitors of SIRT1, SIRT3 and SIRT6 deacetylase activity and of SIRT6 depalmitoylase activity, tested on the recombinant proteins. The standard deviation was always ≤10%.

Compound	% Inhibition SIRT1	% Inhibition SIRT3	% Inhibition SIRT6 (Deacetylase)	% Inhibition SIRT6 (Depalmiotoylase)
**3a**	86	6	42	ni
**3d**	99	23	15	ni
**5a**	100	36	ni	ni
**5d**	92	24	ni	ni
**3b**	98	14	ni	ni
**3c**	100	20	7	ni

ni, no inhibition.

## Data Availability

Data are available upon request to the corresponding authors.

## Supplementary Materials

The following supporting information can be downloaded at: https://www.mdpi.com/article/10.3390/ijms252011084/s1.
