# Supplementary material for: Discovery of Novel Thiazole-Based SIRT2 Inhibitors as Anticancer Agents: Molecular Modeling, Chemical Synthesis and Biological Assays
Source: Int J Mol Sci. 2024 Oct 15;25(20):11084. doi: 10.3390/ijms252011084 (PMC11508362; doi:10.3390/ijms252011084)
Supplement: Supplementary file 1 [file ijms-25-11084-s001.zip › ijms-3231462-supplementary.pdf]

SUPPORTING INFORMATION

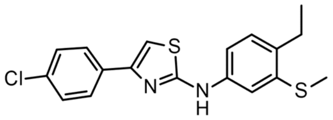

T1

SIRT2 inhibition = 100 %

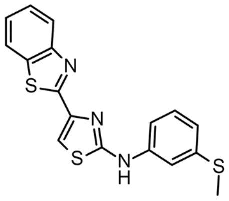

T2

SIRT2 inhibition = 72 %

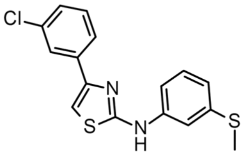

T3

SIRT2 inhibition = 60%

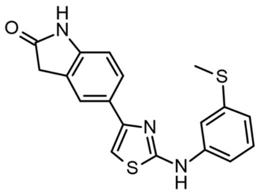

T4

SIRT2 inhibition = 60%

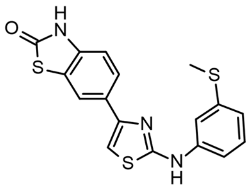

T5

SIRT2 inhibition = 76 %

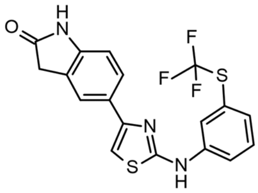

T6

SIRT2 inhibition = 92%

Figure S1. Chemical structure and biological activity as SIRT2Is of the previously developed **T1**-**T6**. Percentage values of SIRT2 inhibition have been determined at 150μM [1].

Table S1. Chemical structure of SirReal2 analogues **1**-**25** and biological data as IC_{50} values of SIRT2 inhibition [2].

Compound	Chemical Structure	IC_{50} (μM)
**1**		3.8
**2**		16.8
**3**		1.6
**4**		3.4

**5**	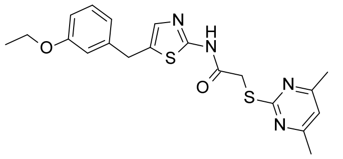	1.3
**6**	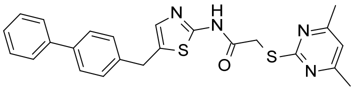	164.5
**7**	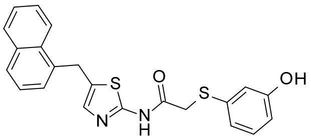	143.0
**8**	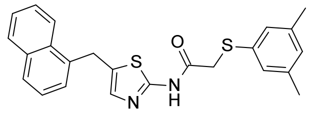	207.0
**9**	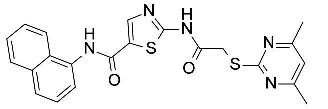	33.0
**SirReal2**	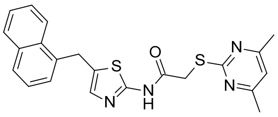	0.4
**10**	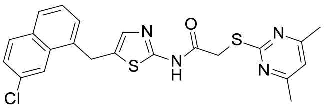	0.2
**11**	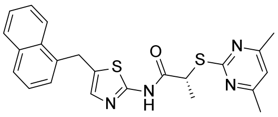	9.8
**12**	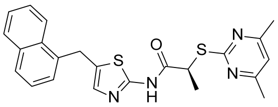	0.3
**13**	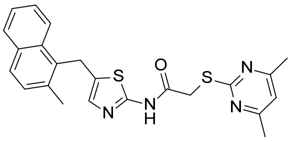	0.3
**14**	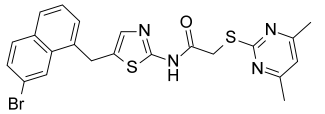	0.2
**15**	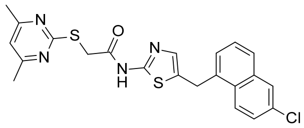	0.5
**16**	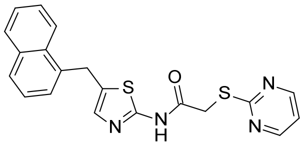	2.3

**17**	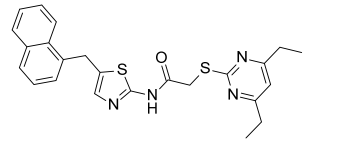	45.6
**18**	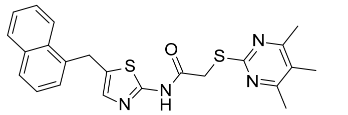	15.0
**19**	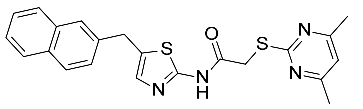	65.0
**20**	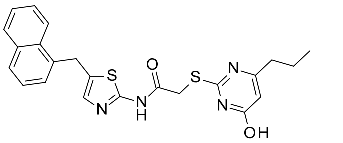	127.2
**21**	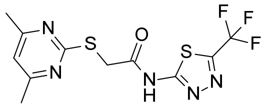	502.8
**22**	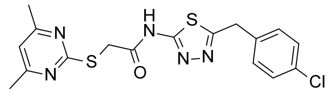	30.9
**23**	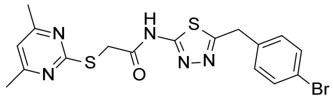	167.7
**24**	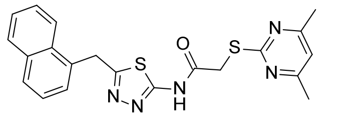	1.9
**25**	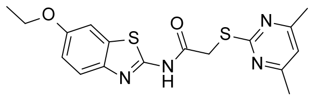	207.0

Table S2. The calculated Glob-Prod values, based on the MIFs H, N1, DRY, O, are reported (SirReal2 analogues **1**-**25** as candidates, being **SirReal2** the template). The most promising **14, 10, 12** and the less ones (**7**, **8**) are depicted in cyan and grey, respectively.

SirReal2 analogues	IC_{50} μM	Glob-Prod	H	N1	DRY	O
**14**	0.21	0.6632	0.7527	0.5994	0.4881	0.8455
**10**	0.18	0.6234	0.6376	0.6298	0.3229	0.8352
**12**	0.26	0.6135	0.7125	0.5417	0.3799	0.7481
**25**	207	0.6127	0.6064	0.5539	0.2910	0.7393
**20**	127.2	0.6079	0.7283	0.5402	0.3989	0.2703
**1**	3.75	0.5884	0.6542	0.5720	0.3639	0.7867
**3**	1.64	0.5866	0.6662	0.5897	0.3948	0.7855
**2**	16.8	0.5841	0.6115	0.5521	0.2931	1.1602
**16**	2.34	0.5820	0.6437	0.5603	0.4089	0.8005
**4**	3.4	0.5787	0.5962	0.6131	0.2943	0.7821
**18**	15	0.5582	0.6459	0.4538	0.3965	0.7268

**13**	0.31	0.5547	0.6260	0.4829	0.3454	0.6844
**17**	45.6	0.5417	0.6558	0.5576	0.3720	0.6988
**22**	30.9	0.5398	0.6447	0.4560	0.2511	0.7313
**24**	1.89	0.5391	0.6847	0.3960	0.3120	0.7242
**21**	502.8	0.5376	0.5582	0.4756	0.2353	0.6924
**19**	65	0.5371	0.6554	0.5645	0.3263	0.6764
**5**	1.33	0.5306	0.6113	0.5609	0.3004	0.7700
**15**	0.48	0.5285	0.6397	0.6048	0.3408	0.7376
**6**	164.5	0.5284	0.5616	0.6331	0.2666	0.8282
**9**	33	0.5255	0.5875	0.4747	0.2681	0.6234
**23**	167.7	0.5128	0.6015	0.5168	0.2546	0.7395
**11**	9.77	0.5101	0.6866	0.5235	0.3810	0.7129
**8**	207	0.4999	0.6669	0.3703	0.3271	0.6215
**7**	143	0.4256	0.6239	0.4046	0.3647	0.2990

Table S3. Ten top scored docking positioning of **SirReal2** (cyan) and of the related analogues (**1**-**25**; Comp.) based on molecular docking calculations at the 4RMG PDB code (MOE software). The predicted ΔG value of each protein-ligand complex has been reported, as calculated in terms of final scoring function (S, as Kcal/mol). The corresponding SIRT2 IC_{50} values (μM) are reported [3].

Comp.	S	E_conf	E_place	E_score1	E_refine	E_score2	IC_{50}
**1**	-9.67	-33.14	-22.71	-8.41	-8.31	-9.67	
**1**	-9.07	-47.82	-23.28	-9.88	-26.13	-9.07	
**1**	-8.98	-44.93	-33.67	-10.83	-23.74	-8.98	
**1**	-8.93	-41.25	-28.15	-8.00	-23.21	-8.93	
**1**	-8.62	-48.54	-27.48	-7.83	-29.61	-8.62	3.8
**1**	-7.95	-42.35	-25.65	-9.61	-5.74	-7.95	
**1**	-7.82	-48.34	-18.78	-9.14	-19.53	-7.82	
**1**	-7.78	-39.44	-18.43	-8.50	-13.83	-7.78	
**1**	-7.37	-49.10	-20.03	-8.10	-15.20	-7.37	
**1**	-7.28	-47.31	-21.04	-8.28	-16.68	-7.28	
**2**	-8.11	-50.79	-25.37	-9.35	-26.17	-8.11	
**2**	-8.08	-39.18	-19.68	-9.58	-7.56	-8.08	
**2**	-7.47	-51.06	-17.81	-8.79	-29.08	-7.47	
**2**	-7.47	-51.06	-32.62	-8.69	-29.08	-7.47	
**2**	-7.31	-47.12	-18.68	-8.45	-17.80	-7.31	
**2**	-7.15	-43.67	-22.31	-8.88	-13.18	-7.15	16.8
**2**	-7.06	-50.58	-21.45	-8.59	-17.20	-7.06	
**2**	-6.97	-48.36	-33.72	-11.22	-12.21	-6.97	
**2**	-6.94	-45.44	-22.97	-9.57	-3.62	-6.94	
**2**	-6.94	-45.76	-21.30	-9.66	-14.80	-6.94	
**3**	-10.59	-18.95	-31.49	-10.14	7.02	-10.59	
**3**	-9.56	-42.20	-20.30	-8.66	-19.23	-9.56	
**3**	-9.35	-38.92	-17.51	-8.23	-24.73	-9.35	
**3**	-9.32	-42.57	-26.54	-9.09	-25.19	-9.32	

**3**	-9.00	-45.25	-38.77	-11.14	-28.27	-9.00	1.6
**3**	-8.89	-38.57	-30.99	-9.45	-11.47	-8.89	
**3**	-8.49	-32.01	-21.05	-7.91	-2.97	-8.49	
**3**	-7.97	-49.66	-24.32	-8.85	-20.56	-7.97	
**3**	-7.76	-45.60	-24.98	-9.42	-17.94	-7.76	
**3**	-7.65	-29.20	-25.32	-8.54	-3.50	-7.65	
**4**	-9.63	-39.60	-30.85	-9.42	-18.68	-9.63	3.4
**4**	-8.90	-39.00	-29.06	-8.78	-22.99	-8.90	
**4**	-8.29	-33.77	-22.10	-9.79	13.70	-8.29	
**4**	-8.25	-45.45	-24.69	-8.94	-12.47	-8.25	
**4**	-8.23	-53.18	-24.09	-9.86	-17.01	-8.23	
**4**	-8.22	-37.37	-19.39	-8.67	-17.83	-8.22	
**4**	-8.12	-45.54	-27.44	-9.09	-8.77	-8.12	
**4**	-8.01	-51.08	-24.61	-9.61	-13.96	-8.01	
**4**	-8.01	-42.84	-23.68	-9.13	-4.98	-8.01	
**4**	-7.86	-44.96	-29.91	-9.63	-14.88	-7.86	
**5**	-8.90	6.80	-21.21	-8.98	6.99	-8.90	1.3
**5**	-8.68	-36.14	-31.69	-8.89	-15.42	-8.68	
**5**	-7.72	-35.34	-21.94	-8.55	-14.95	-7.72	
**5**	-7.70	-43.66	-24.92	-8.88	-18.86	-7.70	
**5**	-7.63	-40.67	-19.46	-7.97	-13.82	-7.63	
**5**	-7.58	-38.93	-25.18	-8.77	-25.61	-7.58	
**5**	-7.18	-38.17	-25.14	-8.29	-25.10	-7.18	
**5**	-7.11	-38.90	-25.98	-9.98	-16.35	-7.11	
**5**	-7.11	-47.61	-25.54	-8.93	-15.38	-7.11	
**5**	-6.85	-43.69	-15.84	-8.55	-22.71	-6.85	
**6**	-10.22	-18.35	-20.86	-8.01	-29.28	-10.22	164.5
**6**	-10.18	-14.67	-19.31	-8.80	-25.04	-10.18	
**6**	-10.13	-27.60	-19.48	-8.35	-20.27	-10.13	
**6**	-10.12	-20.25	-23.29	-7.79	-27.38	-10.12	
**6**	-10.00	-7.38	-16.88	-9.50	51.62	-10.00	
**6**	-9.59	-17.90	-20.50	-7.95	-20.13	-9.59	
**6**	-9.42	-19.63	-23.01	-8.38	-19.31	-9.42	
**6**	-9.34	-2.36	-24.53	-9.18	-11.11	-9.34	
**6**	-9.27	-17.55	-23.86	-11.61	-14.93	-9.27	
**6**	-9.18	-14.77	-24.07	-7.69	-17.06	-9.18	
**7**	-10.69	17.32	-27.43	-8.94	-18.41	-10.69	143.0
**7**	-10.68	34.81	-23.62	-8.66	10.19	-10.68	
**7**	-10.56	21.46	-36.21	-8.86	-14.00	-10.56	
**7**	-10.39	28.12	-22.67	-8.71	9.12	-10.39	
**7**	-10.24	14.32	-32.89	-9.63	-4.33	-10.24	
**7**	-10.15	24.50	-36.02	-11.18	-13.70	-10.15	
**7**	-10.00	17.46	-33.53	-8.89	8.27	-10.00	
**7**	-9.97	16.76	-28.32	-9.20	-14.58	-9.97	
**7**	-9.69	9.71	-19.71	-9.45	-16.39	-9.69	
**7**	-9.40	20.04	-27.08	-9.08	-23.48	-9.40	

**8**	-11.76	40.10	-29.17	-7.82	-1.20	-11.76	
**8**	-11.70	42.57	-31.32	-7.85	-13.99	-11.70	
**8**	-11.56	41.74	-28.04	-9.08	-6.95	-11.56	
**8**	-10.97	38.20	-32.72	-8.48	-14.65	-10.97	
**8**	-10.47	41.36	-26.85	-8.17	-15.62	-10.47	207.0
**8**	-10.34	41.60	-28.26	-9.84	3.77	-10.34	
**8**	-10.25	37.34	-27.38	-9.17	-8.89	-10.25	
**8**	-10.07	59.85	-28.97	-7.64	17.55	-10.07	
**8**	-9.94	42.90	-14.28	-8.52	-21.59	-9.94	
**8**	-9.53	49.67	-24.20	-8.90	13.89	-9.53	
**9**	-8.30	-51.11	-17.50	-7.62	-21.91	-8.30	
**9**	-8.23	-44.79	-15.36	-7.44	-3.59	-8.23	
**9**	-8.22	-39.40	-20.71	-10.25	-6.60	-8.22	
**9**	-8.20	-57.57	-13.90	-9.72	-15.56	-8.20	
**9**	-8.11	-52.12	-21.22	-7.58	-25.91	-8.11	33.0
**9**	-7.86	-54.24	-17.89	-9.91	-24.28	-7.86	
**9**	-7.85	-47.13	-23.13	-7.84	-21.84	-7.85	
**9**	-7.83	-54.96	-28.45	-10.56	-28.84	-7.83	
**9**	-7.75	-53.55	-18.67	-9.83	7.02	-7.75	
**9**	-7.43	-50.09	-20.27	-7.41	-22.15	-7.43	
**10**	-10.66	-28.61	-30.22	-10.07	-27.02	-10.66	
**10**	-10.46	-32.67	-34.68	-8.29	-25.00	-10.46	
**10**	-10.30	-21.69	-16.97	-7.79	-17.49	-10.30	
**10**	-10.13	-33.22	-24.64	-7.99	-25.03	-10.13	
**10**	-9.66	-36.04	-24.08	-8.05	-12.08	-9.66	0.8
**10**	-9.60	-26.21	-28.93	-10.14	-10.58	-9.60	
**10**	-9.31	-29.25	-28.94	-8.10	22.98	-9.31	
**10**	-9.22	-10.19	-22.79	-8.56	34.63	-9.22	
**10**	-8.85	-23.05	-33.57	-8.64	10.68	-8.85	
**10**	-8.67	-4.62	-18.99	-7.95	46.39	-8.67	
**11**	-10.46	-20.32	-29.20	-7.54	-16.19	-10.46	
**11**	-10.21	-23.70	-18.64	-7.49	-23.73	-10.21	
**11**	-10.19	-14.39	-33.46	-8.21	-15.52	-10.19	
**11**	-10.11	5.09	-30.56	-8.62	-16.71	-10.11	
**11**	-9.96	-20.59	-42.50	-10.26	-15.00	-9.96	
**11**	-9.66	-8.42	-38.59	-7.51	7.88	-9.66	9.8
**11**	-9.57	6.02	-25.98	-9.19	4.59	-9.57	
**11**	-9.28	-2.70	-25.65	-7.72	10.88	-9.28	
**11**	-9.21	-14.74	-29.38	-9.52	8.34	-9.21	
**11**	-9.19	-7.44	-38.27	-9.28	-0.68	-9.19	
**12**	-10.22	-4.92	-30.91	-7.59	-7.25	-10.22	
**12**	-9.31	-6.90	-23.26	-7.07	13.25	-9.31	
**12**	-9.29	-15.47	-22.03	-7.03	-8.69	-9.29	
**12**	-9.16	-6.82	-27.70	-7.18	13.10	-9.16	0.3
**12**	-9.12	-10.84	-30.69	-8.32	-23.02	-9.12	
**12**	-9.10	-12.12	-27.52	-6.87	6.53	-9.10	

**12**	-8.74	-20.55	-18.41	-7.06	-17.38	-8.74	
**12**	-8.65	-9.48	-38.06	-8.91	-5.04	-8.65	
**12**	-8.58	-1.58	-22.60	-8.94	11.91	-8.58	
**12**	-8.47	-12.03	-12.91	-6.88	70.46	-8.47	
**13**	-10.91	-26.43	-23.87	-8.22	-28.91	-10.91	0.3
**13**	-10.77	-17.63	-31.27	-10.08	-24.70	-10.77	
**13**	-10.60	-15.70	-34.52	-10.09	-25.25	-10.60	
**13**	-10.54	-13.15	-36.52	-11.10	-18.35	-10.54	
**13**	-10.33	-19.02	-45.51	-8.86	-10.11	-10.33	
**13**	-10.05	-23.56	-31.95	-10.95	-7.17	-10.05	
**13**	-9.88	-13.31	-30.77	-9.68	-11.84	-9.88	
**13**	-9.61	-18.01	-34.36	-7.82	-12.85	-9.61	
**13**	-9.58	-15.70	-27.56	-9.47	-18.51	-9.58	
**13**	-9.58	-18.13	-29.91	-7.29	-16.46	-9.58	
**14**	-10.99	-31.43	-35.45	-8.84	-23.67	-10.99	0.2
**14**	-10.65	-27.40	-32.78	-8.01	-27.44	-10.65	
**14**	-10.02	-27.00	-25.33	-9.02	26.97	-10.02	
**14**	-9.98	-27.56	-28.37	-12.21	-16.18	-9.98	
**14**	-9.96	-16.63	-30.10	-7.52	-19.10	-9.96	
**14**	-9.76	-29.42	-22.86	-7.20	-17.45	-9.76	
**14**	-9.50	-23.15	-28.04	-10.28	-3.82	-9.50	
**14**	-9.33	-28.73	-31.81	-7.03	0.02	-9.33	
**14**	-9.10	-16.94	-32.08	-8.82	12.21	-9.10	
**14**	-9.05	-13.66	-25.66	-8.17	40.98	-9.05	
**15**	-10.62	-25.98	-37.63	-9.07	-12.33	-10.62	0.5
**15**	-10.19	-33.92	-38.87	-9.74	-33.18	-10.19	
**15**	-9.92	-24.02	-30.04	-8.06	-18.13	-9.92	
**15**	-9.46	-27.64	-26.48	-8.00	-22.03	-9.46	
**15**	-9.41	-21.72	-23.35	-8.52	6.54	-9.41	
**15**	-9.35	-15.39	-16.88	-8.44	-13.70	-9.35	
**15**	-9.27	-20.00	-22.02	-8.15	-13.45	-9.27	
**15**	-9.03	-32.39	-20.50	-8.04	-19.48	-9.03	
**15**	-8.90	-29.47	-20.08	-8.36	-20.42	-8.90	
**15**	-8.88	-17.04	-29.46	-8.40	13.58	-8.88	
**16**	-9.13	-34.33	-25.15	-8.53	-11.09	-9.13	2.3
**16**	-9.05	-32.04	-38.16	-11.21	-25.26	-9.05	
**16**	-8.99	-35.37	-26.46	-10.72	-21.75	-8.99	
**16**	-8.85	-35.06	-34.82	-9.15	-31.23	-8.85	
**16**	-8.82	-25.24	-35.43	-9.24	-20.61	-8.82	
**16**	-8.77	-29.40	-29.56	-9.35	-14.18	-8.77	
**16**	-8.73	-15.56	-29.59	-10.17	-0.78	-8.73	
**16**	-8.67	-28.91	-39.32	-10.06	16.03	-8.67	
**16**	-8.42	-35.42	-27.64	-9.16	-14.67	-8.42	
**16**	-8.39	-29.55	-23.41	-9.17	-25.80	-8.39	
**17**	-10.91	-28.84	-29.22	-9.09	-23.50	-10.91	
**17**	-10.76	18.63	-29.56	-9.57	40.61	-10.76	

**17**	-10.75	-15.55	-36.50	-8.80	4.21	-10.75	45.6
**17**	-10.31	-31.65	-16.68	-7.00	-9.12	-10.31	
**17**	-10.25	-31.45	-31.63	-8.04	-10.43	-10.25	
**17**	-10.02	-29.20	-21.05	-7.89	-17.76	-10.02	
**17**	-9.76	-25.70	-20.55	-8.42	-18.08	-9.76	
**17**	-9.69	-24.00	-20.87	-8.06	-4.48	-9.69	
**17**	-9.63	-20.56	-27.18	-8.70	-8.85	-9.63	
**17**	-9.59	-28.56	-23.29	-7.43	-7.07	-9.59	
**18**	-10.55	-21.40	-19.65	-7.36	-24.86	-10.55	15.0
**18**	-10.32	-15.39	-24.67	-9.19	-11.78	-10.32	
**18**	-10.31	-19.04	-38.22	-7.20	-8.04	-10.31	
**18**	-10.09	-26.46	-24.70	-9.74	-24.55	-10.09	
**18**	-10.07	-22.27	-31.13	-7.52	-16.64	-10.07	
**18**	-9.59	-18.01	-28.14	-12.14	-9.53	-9.59	
**18**	-9.40	-11.78	-24.89	-7.62	18.99	-9.40	
**18**	-9.16	-10.36	-33.14	-7.56	-2.37	-9.16	
**18**	-8.95	-12.57	-22.25	-9.13	-6.04	-8.95	
**18**	-8.75	-17.68	-25.67	-7.42	-19.32	-8.75	
**19**	-9.90	-23.21	-38.43	-10.12	-22.45	-9.90	65.0
**19**	-9.83	-31.95	-21.35	-8.97	-20.54	-9.83	
**19**	-9.78	-35.44	-25.33	-8.27	-8.54	-9.78	
**19**	-9.71	-35.48	-24.43	-9.25	-15.01	-9.71	
**19**	-9.69	-36.12	-25.51	-9.49	3.06	-9.69	
**19**	-9.58	-33.93	-29.45	-8.25	-29.69	-9.58	
**19**	-9.19	-2.26	-26.71	-8.62	-8.24	-9.19	
**19**	-9.02	-7.93	-28.26	-9.94	11.89	-9.02	
**19**	-9.02	5.64	-31.16	-8.93	7.72	-9.02	
**19**	-8.96	-27.57	-29.48	-8.62	-9.24	-8.96	
**20**	-10.35	-74.53	-26.57	-7.86	1.19	-10.35	127.2
**20**	-10.18	-65.63	-29.59	-7.82	-9.48	-10.18	
**20**	-10.14	-73.87	-17.73	-7.24	-13.59	-10.14	
**20**	-9.82	-71.46	-26.72	-10.49	-16.95	-9.82	
**20**	-9.67	-75.33	-28.02	-8.31	-13.02	-9.67	
**20**	-9.61	-74.15	-22.45	-9.43	-25.28	-9.61	
**20**	-9.25	-59.26	-20.91	-7.63	-1.74	-9.25	
**20**	-9.04	-76.87	-21.08	-10.53	-12.81	-9.04	
**20**	-8.88	-70.92	-34.87	-9.25	11.89	-8.88	
**20**	-8.87	-74.11	-22.13	-7.05	-15.28	-8.87	
**21**	-6.58	-45.20	-24.12	-11.02	-21.32	-6.58	502.8
**21**	-6.29	-39.15	-29.41	-9.84	-18.58	-6.29	
**21**	-6.26	-41.30	-24.15	-10.33	-22.69	-6.26	
**21**	-6.14	-39.82	-14.33	-9.67	-8.98	-6.14	
**21**	-6.00	-35.69	-23.61	-11.13	-15.88	-6.00	
**21**	-5.89	-36.05	-22.51	-9.69	-9.92	-5.89	
**21**	-5.71	-38.85	-17.13	-9.47	-16.89	-5.71	
**21**	-5.69	-39.02	-21.83	-9.26	-5.04	-5.69	

**21**	-5.57	-28.82	-19.60	-10.27	7.18	-5.57	
**21**	-5.44	-36.34	-15.30	-9.19	5.58	-5.44	
**22**	-7.95	-32.55	-29.28	-10.16	-21.61	-7.95	30.9
**22**	-7.87	-38.87	-21.00	-9.18	-6.97	-7.87	
**22**	-7.85	-47.69	-25.33	-10.18	-19.41	-7.85	
**22**	-7.79	-44.52	-19.72	-10.01	-6.86	-7.79	
**22**	-7.60	-38.21	-18.80	-9.38	-10.70	-7.60	
**22**	-7.17	-25.08	-32.53	-10.56	-7.98	-7.17	
**22**	-6.86	-41.34	-21.44	-8.98	-20.05	-6.86	
**22**	-6.82	-31.40	-30.84	-10.56	-17.17	-6.82	
**22**	-6.57	-36.52	-24.68	-9.23	-15.91	-6.57	
**22**	-6.55	-37.88	-20.82	-9.59	-3.95	-6.55	
**23**	-8.23	-37.35	-21.38	-8.96	-10.43	-8.23	167.7
**23**	-7.97	-46.77	-25.23	-10.13	-16.45	-7.97	
**23**	-7.94	-37.12	-21.08	-9.48	-3.17	-7.94	
**23**	-7.88	-31.94	-29.36	-10.43	-21.63	-7.88	
**23**	-7.85	-43.82	-19.41	-10.10	-3.43	-7.85	
**23**	-7.65	-43.12	-25.89	-9.11	-14.54	-7.65	
**23**	-7.12	-40.03	-20.78	-8.92	-18.77	-7.12	
**23**	-7.11	-23.90	-27.97	-9.56	-8.30	-7.11	
**23**	-6.98	-40.28	-19.99	-10.26	-19.39	-6.98	
**23**	-6.84	-30.82	-30.76	-10.63	-17.21	-6.84	
**24**	-9.80	-0.29	-25.32	-8.91	1.68	-9.80	1.9
**24**	-9.50	48.08	-27.65	-8.34	13.29	-9.50	
**24**	-9.43	-23.85	-21.72	-8.05	-27.97	-9.43	
**24**	-9.29	-22.93	-34.90	-9.29	-25.11	-9.29	
**24**	-9.17	-15.16	-25.48	-9.42	-19.48	-9.17	
**24**	-8.80	-24.91	-27.72	-8.60	-25.22	-8.80	
**24**	-8.69	-22.09	-23.57	-8.68	-20.83	-8.69	
**24**	-8.63	-22.20	-28.80	-9.01	-18.57	-8.63	
**24**	-8.43	-20.39	-31.61	-8.42	-7.71	-8.43	
**24**	-8.20	-26.04	-30.19	-10.84	-15.99	-8.20	
**25**	-8.42	-48.46	-32.63	-9.26	-12.02	-8.42	207.0
**25**	-8.33	-51.86	-29.86	-9.25	-10.64	-8.33	
**25**	-8.14	-38.20	-28.05	-9.32	-6.31	-8.14	
**25**	-7.53	-48.72	-27.80	-9.07	-18.61	-7.53	
**25**	-7.53	-45.50	-26.89	-8.80	-6.20	-7.53	
**25**	-7.42	-29.09	-23.50	-9.04	14.37	-7.42	
**25**	-7.13	-45.29	-18.28	-9.55	-11.69	-7.13	
**25**	-6.82	-52.57	-20.75	-8.61	-26.36	-6.82	
**25**	-6.81	-52.80	-23.98	-8.94	-24.14	-6.81	
**25**	-6.75	-48.35	-24.78	-8.79	-21.95	-6.75	
**SirReal2**	-10.30	-24.76	-34.01	-9.05	-25.98	-10.30	
**SirReal2**	-9.94	-30.60	-27.36	-9.06	-9.68	-9.94	
**SirReal2**	-9.62	-32.51	-23.14	-9.69	-24.25	-9.62	
**SirReal2**	-9.55	-24.97	-35.17	-8.26	-8.11	-9.55	

**SirReal2**	-9.53	-28.37	-27.76	-9.42	-24.83	-9.53	0.4
**SirReal2**	-9.36	-18.88	-25.07	-7.77	-7.39	-9.36	
**SirReal2**	-9.11	-26.49	-20.64	-7.53	-17.78	-9.11	
**SirReal2**	-8.90	-33.59	-31.81	-8.74	-3.99	-8.90	
**SirReal2**	-8.80	-27.07	-30.98	-8.49	-8.38	-8.80	
**SirReal2**	-8.61	-28.57	-22.62	-9.01	-2.49	-8.61	

Table S4. Ten top scored docking positioning of the newly developed **3a-3d**,**5a**, **5d** (Comp.) as SIRT2Is via molecular docking calculation at the 4RMG PDB code (MOE software). The predicted ΔG value of each protein-ligand complex has been reported, as calculated in terms of final scoring function (S, as Kcal/mol). The corresponding SIRT2 inhibition percentage at 150 μM (SIRT2 % inhib.) has been added, based on the biochemical assay on the recombinant protein.

Comp.	S	E_conf	E_place	E_score1	E_refine	E_score2	SIRT2 % inhib.
**3a**	-9.49	-4.00	-24.13	-9.00	7.99	-9.49	100
**3a**	-8.97	-6.72	-25.16	-8.64	-5.64	-8.97	
**3a**	-8.87	-9.76	-27.31	-8.71	-3.37	-8.87	
**3a**	-8.66	-30.69	-21.14	-8.73	-13.35	-8.66	
**3a**	-8.60	-20.46	-30.55	-9.37	5.48	-8.60	
**3a**	-8.19	-42.05	-24.38	-7.67	-21.02	-8.19	
**3a**	-8.07	56.26	-21.37	-7.85	57.07	-8.07	
**3a**	-8.03	-37.49	-25.04	-7.99	-16.75	-8.03	
**3a**	-8.01	-31.70	-20.75	-9.77	-24.45	-8.01	
**3a**	-7.90	-5.90	-20.13	-8.78	41.63	-7.90	
**3b**	-8.30	-20.89	-25.28	-10.24	-14.09	-8.30	93
**3b**	-8.22	-23.03	-24.53	-8.76	0.48	-8.22	
**3b**	-8.10	-25.07	-27.85	-9.60	13.78	-8.10	
**3b**	-7.89	-44.48	-32.11	-8.30	-23.87	-7.89	
**3b**	-7.77	-48.38	-15.38	-9.13	-17.83	-7.77	
**3b**	-7.75	-22.64	-24.45	-8.32	-18.06	-7.75	
**3b**	-7.71	-36.56	-28.13	-10.35	-19.01	-7.71	
**3b**	-7.71	-49.66	-23.40	-10.20	-22.64	-7.71	
**3b**	-7.67	-37.83	-20.71	-8.67	-1.02	-7.67	
**3b**	-7.66	-44.99	-24.66	-9.01	-22.18	-7.66	
**3c**	-10.54	-11.65	-16.25	-8.11	-7.13	-10.54	96
**3c**	-9.17	-31.72	-23.43	-8.55	4.63	-9.17	
**3c**	-9.03	-20.46	-23.12	-7.59	12.08	-9.03	
**3c**	-8.87	-20.48	-23.61	-9.49	-6.25	-8.87	
**3c**	-8.81	-49.91	-32.31	-7.64	-23.15	-8.81	
**3c**	-8.47	-44.20	-24.36	-8.12	6.04	-8.47	
**3c**	-8.44	-30.15	-25.69	-9.05	-2.00	-8.44	
**3c**	-8.42	-26.63	-23.86	-8.90	-13.10	-8.42	
**3c**	-8.34	-33.86	-27.31	-7.96	-19.63	-8.34	
**3c**	-7.99	-43.41	-26.57	-8.48	-16.97	-7.99	

**3d**	-9.75	-55.34	-25.82	-9.07	-10.03	-9.75	100
**3d**	-9.67	-38.74	-27.48	-9.65	-8.41	-9.67	
**3d**	-9.09	-53.91	-29.12	-9.92	-9.41	-9.09	
**3d**	-8.79	-53.06	-24.63	-7.82	-2.59	-8.79	
**3d**	-8.77	-45.97	-24.03	-8.40	10.78	-8.77	
**3d**	-8.65	-62.96	-19.84	-8.23	-15.63	-8.65	
**3d**	-8.40	-70.04	-27.94	-8.94	-22.08	-8.40	
**3d**	-8.24	-75.11	-20.66	-8.87	-20.50	-8.24	
**3d**	-8.20	-60.77	-23.28	-8.37	-18.14	-8.20	
**3d**	-8.19	-70.20	-26.06	-8.41	-18.90	-8.19	
**5a**	-9.87	-14.85	-31.34	-8.19	4.18	-9.87	98
**5a**	-8.92	-43.12	-25.30	-11.28	-11.27	-8.92	
**5a**	-8.88	-31.33	-26.42	-8.27	7.30	-8.88	
**5a**	-8.79	-50.56	-21.72	-9.10	-23.82	-8.79	
**5a**	-8.75	-51.11	-22.80	-8.90	-22.99	-8.75	
**5a**	-8.72	-54.22	-24.48	-8.20	-30.24	-8.72	
**5a**	-8.64	-27.51	-21.36	-8.29	3.28	-8.64	
**5a**	-8.53	-36.72	-15.98	-7.95	-12.21	-8.53	
**5a**	-8.34	-18.29	-26.46	-8.43	-10.74	-8.34	
**5a**	-8.29	-37.59	-24.96	-8.59	-6.80	-8.29	
**5d**	-9.28	-50.11	-25.19	-7.07	-5.19	-9.28	93
**5d**	-9.20	-61.99	-24.47	-7.96	3.93	-9.20	
**5d**	-9.15	-62.21	-14.26	-7.26	20.85	-9.15	
**5d**	-9.15	-44.86	-27.73	-6.96	13.84	-9.15	
**5d**	-9.08	-81.31	-12.90	-6.81	-3.44	-9.08	
**5d**	-9.04	-62.88	-23.52	-7.00	-10.59	-9.04	
**5d**	-8.89	-79.69	-20.74	-7.46	-18.72	-8.89	
**5d**	-8.86	-58.53	-24.34	-8.48	-15.17	-8.86	
**5d**	-8.86	-19.61	-16.49	-7.10	59.64	-8.86	
**5d**	-8.68	-80.80	-21.82	-7.30	-20.59	-8.68	

^{1}H Compound 3c

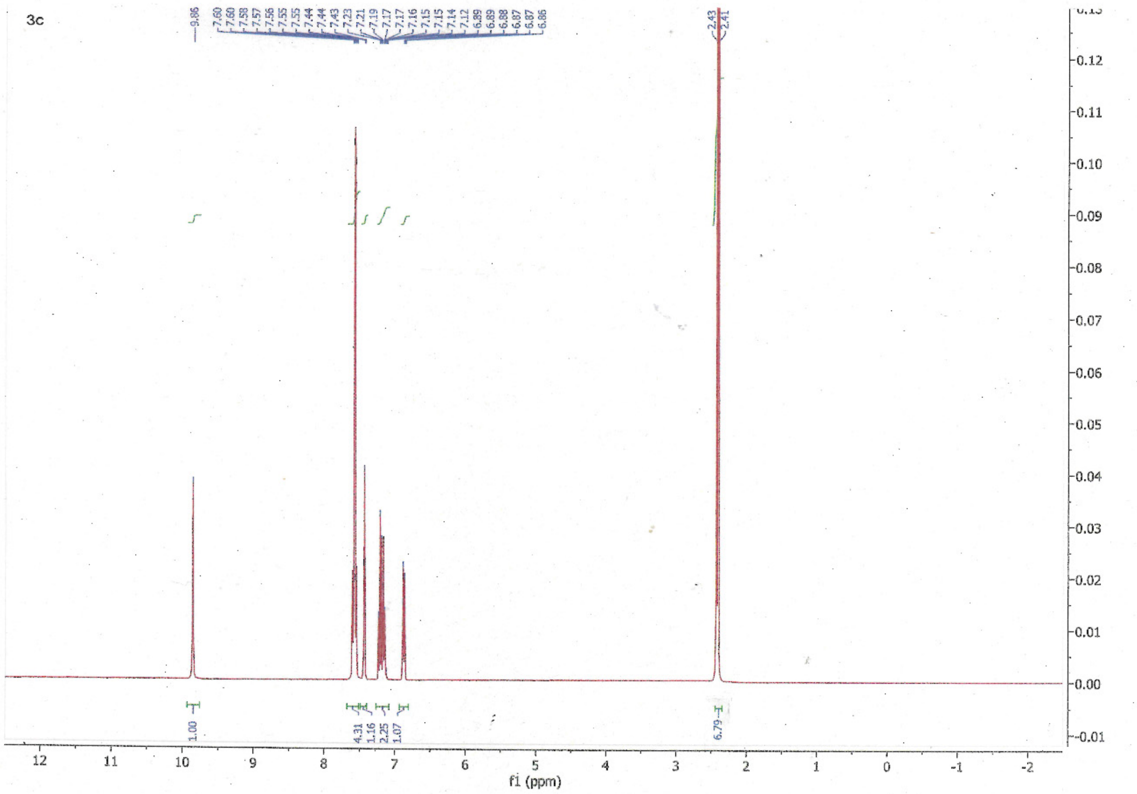

^{13}C Compound 3c

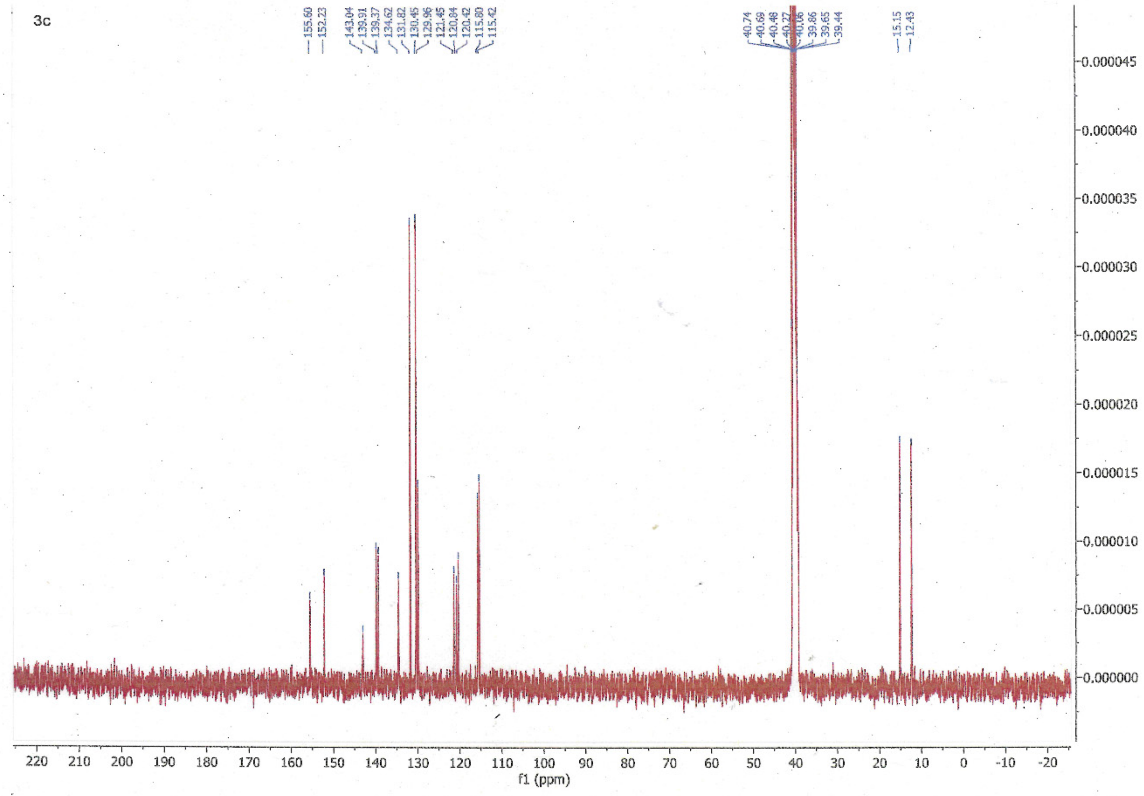

[1]H Compound 5a

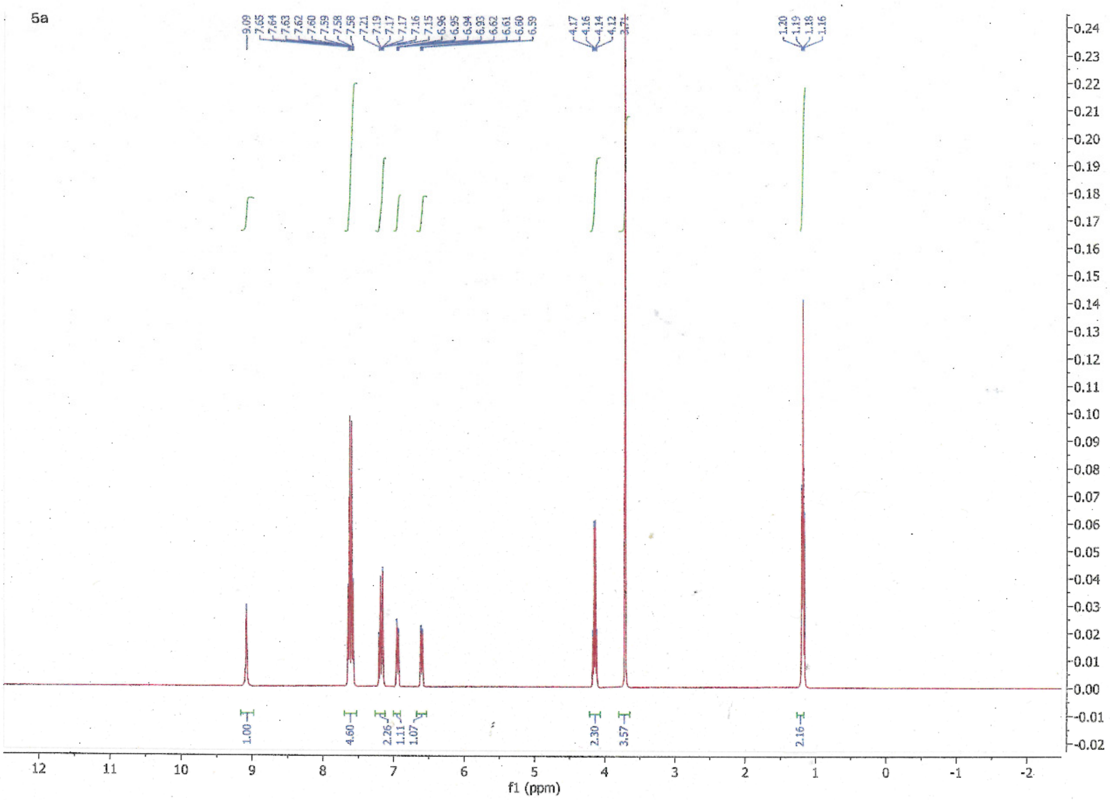

[13]C Compound 5a

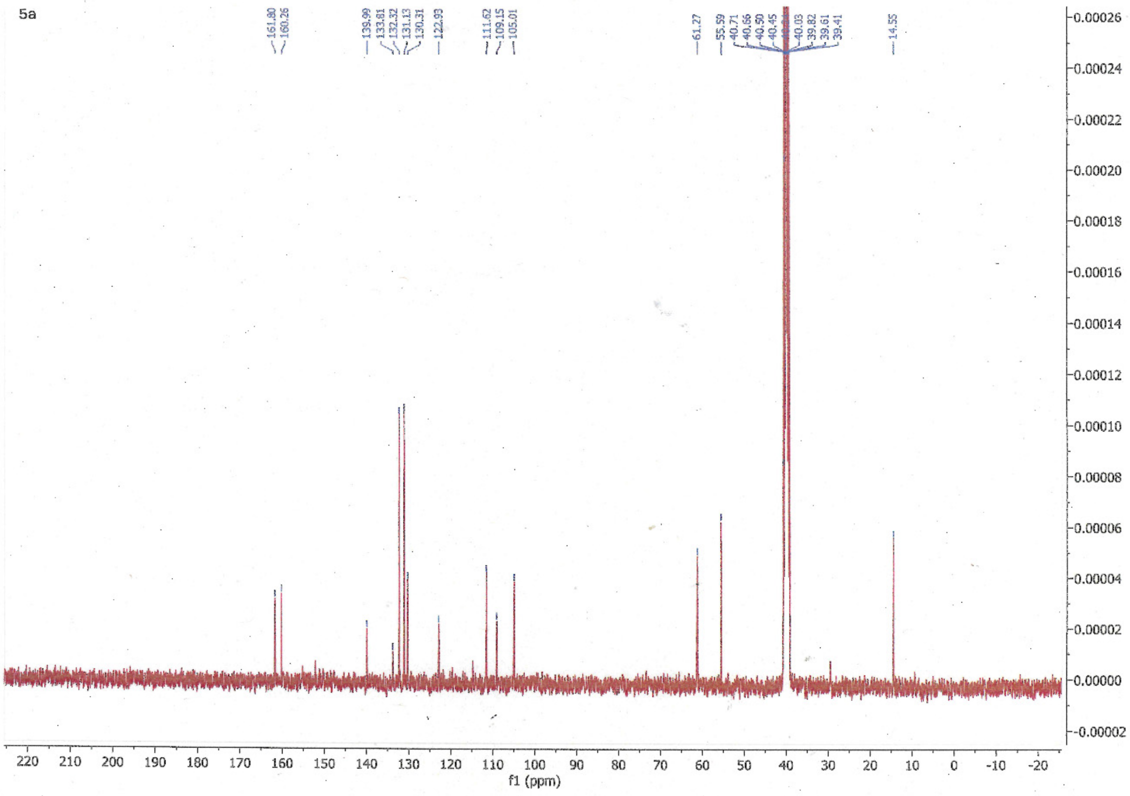

Figure S2. ^{1}H NMR and ^{13}C NMR spectra of compounds **3c** and **5a**

References

1. Abbotto, E.; Casini, B.; Piacente, F.; Scarano, N.; Cerri, E.; Tonelli, M.; Astigiano, C.; Millo, E.; Sturla, L.; Bruzzone, S.; et al. Novel Thiazole-Based SIRT2 Inhibitors Discovered via Molecular Modelling Studies and Enzymatic Assays. *Pharmaceuticals* **2023**, *16*, 1316. https://doi.org/10.3390/ph16091316.
2. Schiedel, M.; Rumpf, T.; Karaman, B.; Lehotzky, A.; Olah, J.; Gerhardt, S.; Ovadi, J.; Sippl, W.; Einsle, O.; Jung, M. Aminothiazoles as potent and selective sirt2 inhibitors: A structure-activity relationship study. *J. Med. Chem.* **2016**, *59*, 1599–1612. https://doi.org/10.1021/acs.jmedchem.5b01517.
3. Fiorentino, F.; Mautone, N.; Menna, M.; D'Acunzo, F.; Mai, A.; Rotili, D. Sirtuin modulators: Past, present, and future perspectives. *Future Med. Chem.* **2022**, *14*, 915–939. https://doi.org/10.4155/fmc-2022-0031.
